# Supplementary material for: A multiomic atlas of the aging hippocampus reveals molecular changes in response to environmental enrichment
Source: Nat Commun. 2024 Jul 16;15:5829. doi: 10.1038/s41467-024-49608-z (PMC11252340; doi:10.1038/s41467-024-49608-z)
Supplement: Supplementary file 1 — Supplementary Information [file 41467_2024_49608_MOESM1_ESM.pdf]

SUPPLEMENTARY FIGURES

This document contains the supplementary figures 1-10 for the manuscript "A MULTIOMIC ATLAS OF THE AGING HIPPOCAMPUS REVEALS MOLECULAR CHANGES IN RESPONSE TO ENVIRONMENTAL ENRICHMENT".

Additionally, the raw sequencing data have been deposited in the European Nucleotide Archive (ENA) under the following accession numbers: PRJEB58981 [https://www.ebi.ac.uk/ena/browser/view/PRJEB58981] (RNA-seq), PRJEB59326 [https://www.ebi.ac.uk/ena/browser/view/PRJEB59326] (EM-seq), PRJEB59328 [https://www.ebi.ac.uk/ena/browser/view/PRJEB59328] (ATAC-seq), PRJEB59330 [https://www.ebi.ac.uk/ena/browser/view/PRJEB59330] (ChIP-seq) and PRJEB59404 [https://www.ebi.ac.uk/ena/browser/view/PRJEB59404] (single cell RNA-seq and ATAC-seq). The raw proteomics data have been deposited in the Proteomics Identification Database (PRIDE) under the accession number PXD045567 [https://www.ebi.ac.uk/pride/archive/projects/PXD045567]. Finally, preprocessed and extended data sets, including chromatin state annotations, preprocessed single cell data (Seurat objects), and the code used in the analyse, are available in a Zenodo repository at https://zenodo.org/doi/10.5281/zenodo.8372431.

SUPPLEMENTARY FIGURES

FIGURE S1

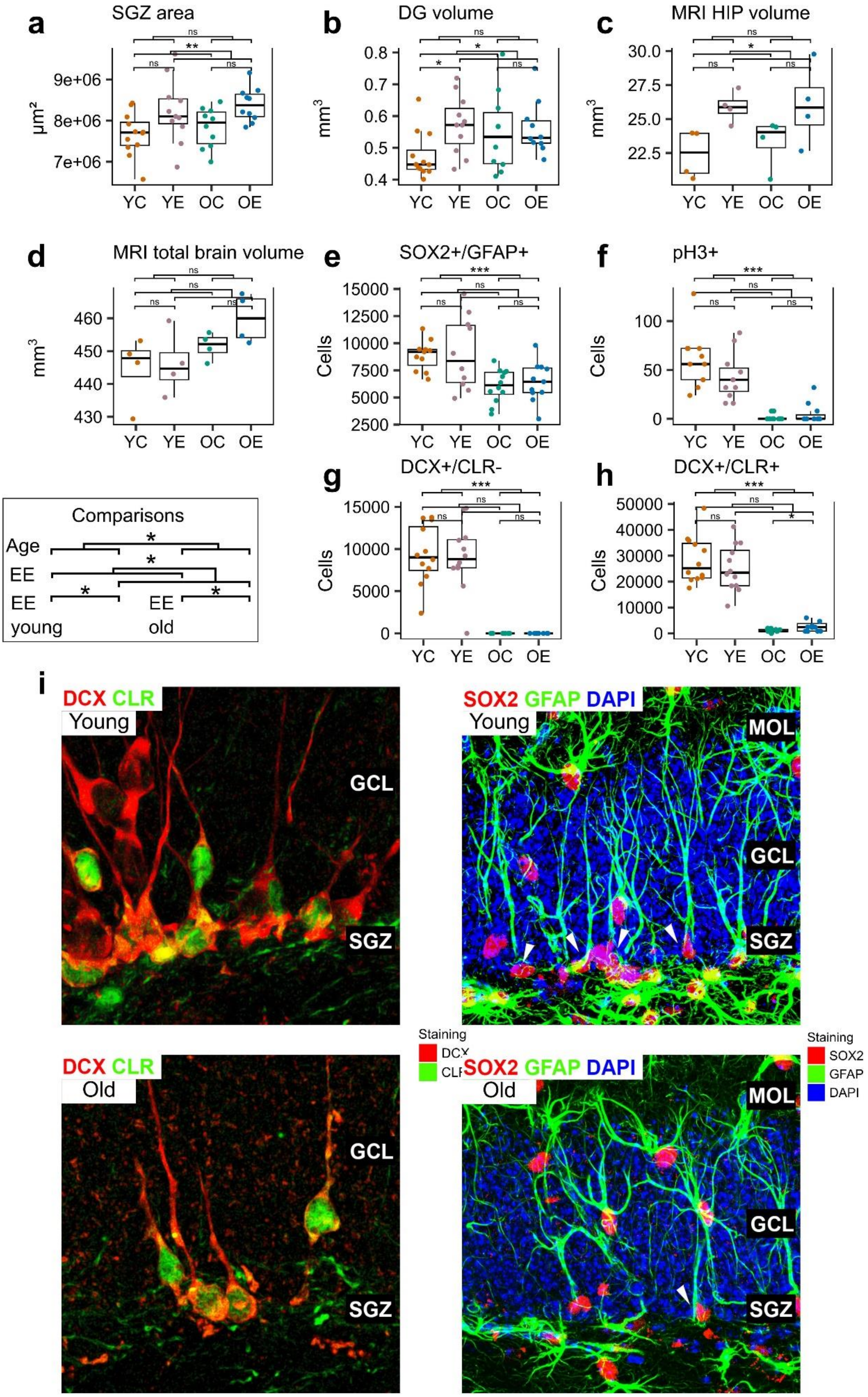
a
SGZ area
µm²
9e+06
8e+06
7e+06
YC YE OC OE
b
DG volume
mm3
YC YE OC OE
c
MRI HIP volume
mm3
YC YE OC OE
d
MRI total brain volume
mm3
YC YE OC OE
e
SOX2+/GFAP+
Cells
YC YE OC OE
f
pH3+
Cells
YC YE OC OE
Comparisons
Age
EE
EE young
EE old
g
DCX+/CLR-
Cells
YC YE OC OE
h
DCX+/CLR+
Cells
YC YE OC OE
i
DCX CLR
Young
Old
GCL
SGZ
SOX2 GFAP DAPI
MOL
Staining
DCX
CLR
SOX2
GFAP
DAPI

FIGURE S1

Fig. S1. Anatomic and cellular alterations in aging and environmental enrichment. a-b, Boxplots showing histological measurements of the SGZ area (**a**) and DG volume (**b**) across study groups. **c-d**, Boxplots indicating MRI volumetric measurements of the hippocampal formation (**c**) and total brain (**d**) across study groups. **e-h**, Boxplots describing cell counts for a series of immunohistochemical markers in the SGZ+GCL hippocampal regions: SOX2+/GFAP+ (**e**, neural stem cells), pH3+ (**f**, mitotic cells), DCX+/CLR- (**g**, immature neurogenic population) and DCX+/CLR+ (**h**, immature neurogenic population). **i**, Representative fluorescence microscopy images of young and old individuals with DCX/CLR and SOX2/GFAP/DAPI staining. $^{*}p < 0.05$, $^{**}p < 0.01$, $^{***}p < 0.001$ for two-sided Wilcoxon rank sum tests. P-values are adjusted for multiple testing within each set of comparisons.

FIGURE S2

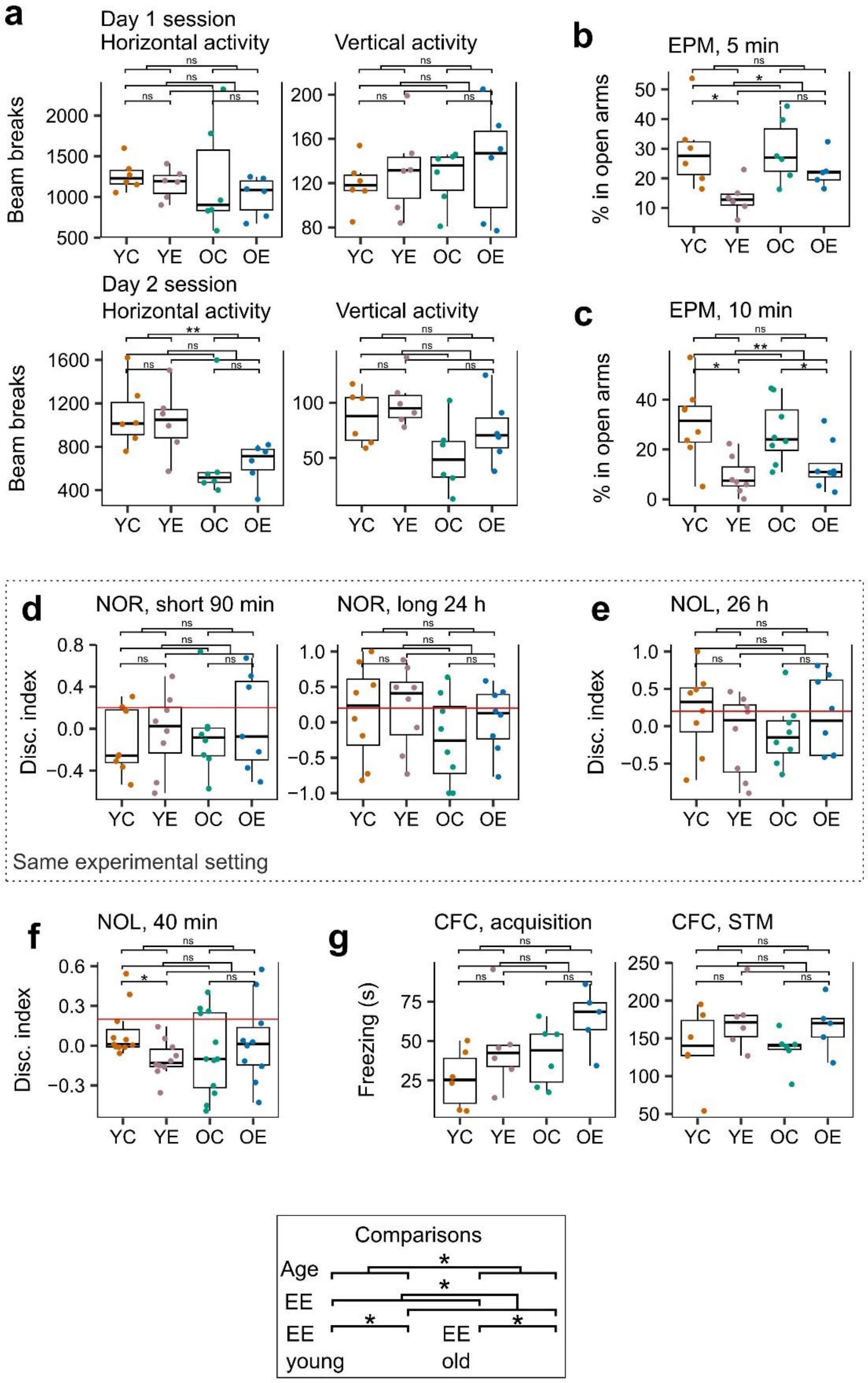

a
Day 1 session
Horizontal activity
Vertical activity
Beam breaks
Day 2 session
Horizontal activity
Vertical activity
b
EPM, 5 min
% in open arms
c
EPM, 10 min
d
NOR, short 90 min
NOR, long 24 h
Disc. index
e
NOL, 26 h
Same experimental setting
f
NOL, 40 min
g
CFC, acquisition
CFC, STM
Freezing (s)
YC YE OC OE
Comparisons
Age
EE
EE young
EE old

FIGURE S2

Fig. S2. Behavioural profiling of the effects of aging and environmental enrichment. **a**, Boxplots showing the actimetry physical activity measurements for day 1 and day 2 sessions in terms of beam break counts in the horizontal and vertical dimensions. **b**, Boxplots describing the elevated plus maze (EPM) test results for the 5 min exploration time setting, showing the percentage time spent in open arms across groups. **c**, Boxplots indicating the EPM test results for the 10 min exploration time setting, showing the percentage time spent in open arms across groups. **d**, Boxplots showing the results for the novel object recognition (NOR) test in terms of discrimination index of time spent exploring Old and New objects estimated as [time N – time O / (time N + time O)] during two consecutive sessions: short-term 90 min and long-term 24 h after training. An horizontal red line indicates DI = 0.2. **e**, Boxplots describing the results for the novel object location (NOL) test performed 90 min after the previous NOL sessions, in terms of discrimination index of time spent exploring Fixed and Moved objects estimated as [time M – time F / (time M + time F)] in a single session of long-term ~26 h after training. An horizontal red line indicates DI = 0.2. **f**, Boxplots indicating the NOL test results for an independent setting consisting of a session 40 min after training with no prior NOR. An horizontal red line indicates DI = 0.2. **g**, Boxplots showing the results for the contextual fear conditioning (CFC) test in terms of freezing time in the different phases: acquisition and short-term. *p < 0.05, **p < 0.01, ***p < 0.001 for two-sided Wilcoxon rank sum tests. P-values are adjusted for multiple testing within each set of comparisons.

FIGURE S3

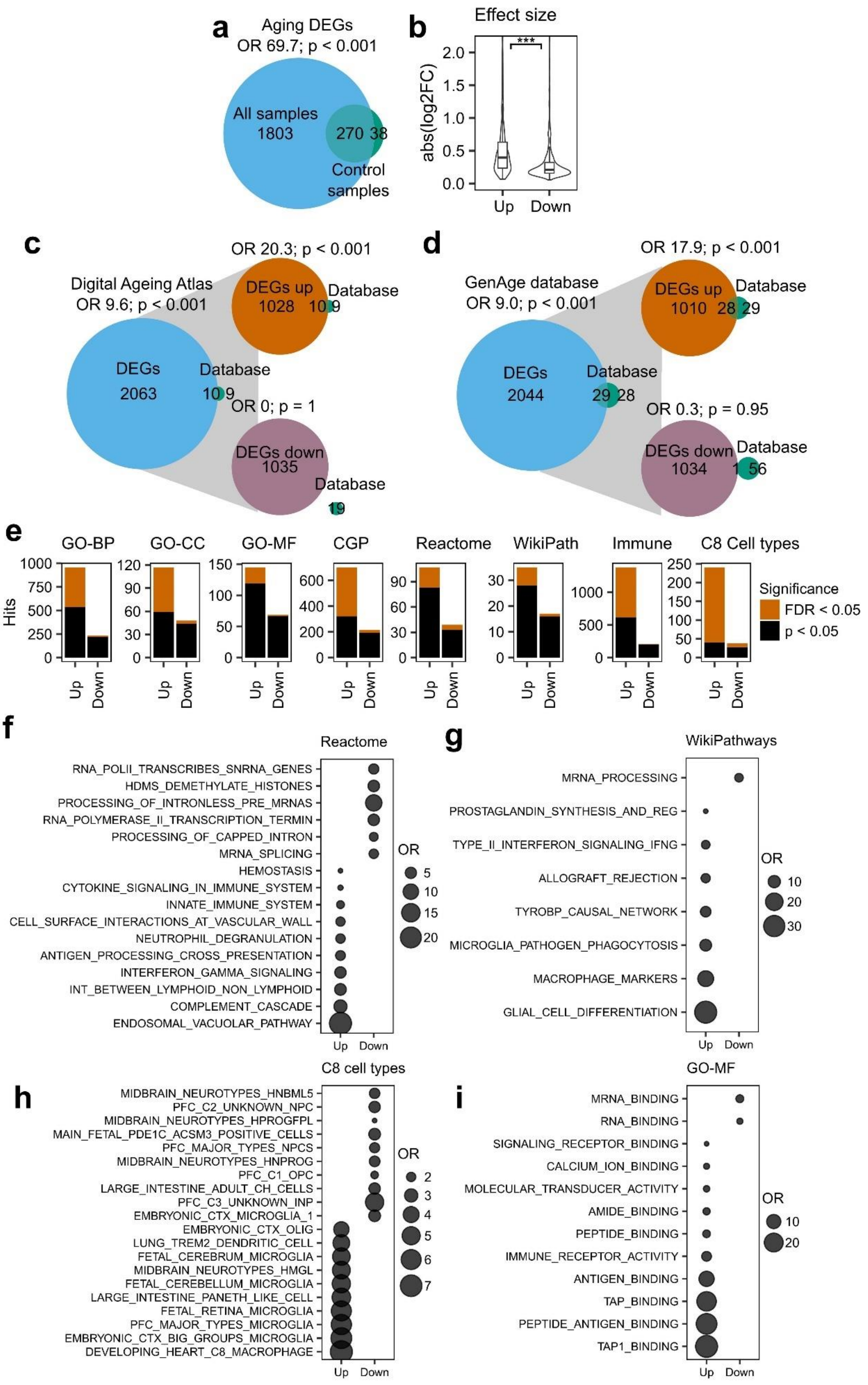

a
Aging DEGs
OR 69.7; p < 0.001
All samples
1803
270 38
Control samples
b
Effect size
abs(log2FC)
***
Up
Down
c
Digital Ageing Atlas
OR 9.6; p < 0.001
DEGs
2063
Database
10 9
OR 20.3; p < 0.001
DEGs up
1028
10 9
Database
OR 0; p = 1
DEGs down
1035
Database
19
d
GenAge database
OR 9.0; p < 0.001
DEGs
2044
Database
29 28
OR 17.9; p < 0.001
DEGs up
1010 28 29
Database
OR 0.3; p = 0.95
DEGs down
1034 1 56
Database
e
GO-BP
GO-CC
GO-MF
CGP
Reactome
WikiPath
Immune
C8 Cell types
Hits
Significance
FDR < 0.05
p < 0.05
f
Reactome
RNA_POLII_TRANSCRIBES_SNRNA_GENES
HDMS_DEMETHYLATE_HISTONES
PROCESSING_OF_INTRONLESS_PRE_MRNAS
RNA_POLYMERASE_II_TRANSCRIPTION_TERMIN
PROCESSING_OF_CAPPED_INTRON
MRNA_SPLICING
HEMOSTASIS
CYTOKINE_SIGNALING_IN_IMMUNE_SYSTEM
INNATE_IMMUNE_SYSTEM
CELL_SURFACE_INTERACTIONS_AT_VASCULAR_WALL
NEUTROPHIL_DEGRANULATION
ANTIGEN_PROCESSING_CROSS_PRESENTATION
INTERFERON_GAMMA_SIGNALING
INT_BETWEEN_LYMPHOID_NON_LYMPHOID
COMPLEMENT_CASCADE
ENDOSOMAL_VACUOLAR_PATHWAY
OR
g
WikiPathways
MRNA_PROCESSING
PROSTAGLANDIN_SYNTHESIS_AND_REG
TYPE_II_INTERFERON_SIGNALING_IFNG
ALLOGRAFT_REJECTION
TYROBP_CAUSAL_NETWORK
MICROGLIA_PATHOGEN_PHAGOCYTOSIS
MACROPHAGE_MARKERS
GLIAL_CELL_DIFFERENTIATION
h
C8 cell types
MIDBRAIN_NEUROTYPES_HNBML5
PFC_C2_UNKNOWN_NPC
MIDBRAIN_NEUROTYPES_HPROGFPL
MAIN_FETAL_PDE1C_ACSM3_POSITIVE_CELLS
PFC_MAJOR_TYPES_NPCS
MIDBRAIN_NEUROTYPES_HNPROG
PFC_C1_OPC
LARGE_INTESTINE_ADULT_CH_CELLS
PFC_C3_UNKNOWN_INP
EMBRYONIC_CTX_MICROGLIA_1
EMBRYONIC_CTX_OLIG
LUNG_TREM2_DENDRITIC_CELL
FETAL_CEREBRUM_MICROGLIA
MIDBRAIN_NEUROTYPES_HMGL
FETAL_CEREBELLUM_MICROGLIA
LARGE_INTESTINE_PANETH_LIKE_CELL
FETAL_RETINA_MICROGLIA
PFC_MAJOR_TYPES_MICROGLIA
EMBRYONIC_CTX_BIG_GROUPS_MICROGLIA
DEVELOPING_HEART_C8_MACROPHAGE
i
GO-MF
MRNA_BINDING
RNA_BINDING
SIGNALING_RECEPTOR_BINDING
CALCIUM_ION_BINDING
MOLECULAR_TRANSDUCER_ACTIVITY
AMIDE_BINDING
PEPTIDE_BINDING
IMMUNE_RECEPTOR_ACTIVITY
ANTIGEN_BINDING
TAP_BINDING
PEPTIDE_ANTIGEN_BINDING
TAP1_BINDING

FIGURE S3

Fig. S3. Functional transcriptomic alterations of aging in the dorsal hippocampus. a, Venn diagram showing the overlap between aging DEGs (FDR < 0.05, two-sided Wald tests) detected using all samples or only control samples. **b**, Violin plots showing the distributions of absolute log2(fold change) for up- and down-regulated DEGs with aging. **c-d**, Venn diagrams indicating the overlap of aging DEGs with canonical aging gene sets from the Digital Ageing Atlas (**c**) and GenAge (**d**) databases. The overlaps are shown both for all DEGs (left plots) and taking into account directionality (right plots). **e**, Bar plots showing the numbers of enriched pathways (p < 0.05 or FDR < 0.05, one-sided Wallenius tests) detected in the gene set enrichment analyses across various databases (Gene Ontology, Chemical and Genetic Perturbation, Reactome, WikiPathways, ImmuneSigDB, C8 cell type signatures) for aging up- and downregulated DEGs. **f-i** Bubble plots indicating the top 10 pathways found enriched (FDR < 0.05, one-sided Wallenius tests) across the Reactome (**f**), WikiPathways (**g**), C8 Cell type signatures (**h**) and Gene Ontology Molecular Function (**i**) databases. The size of the bubbles indicates the odds ratio of enrichment.

FIGURE S4

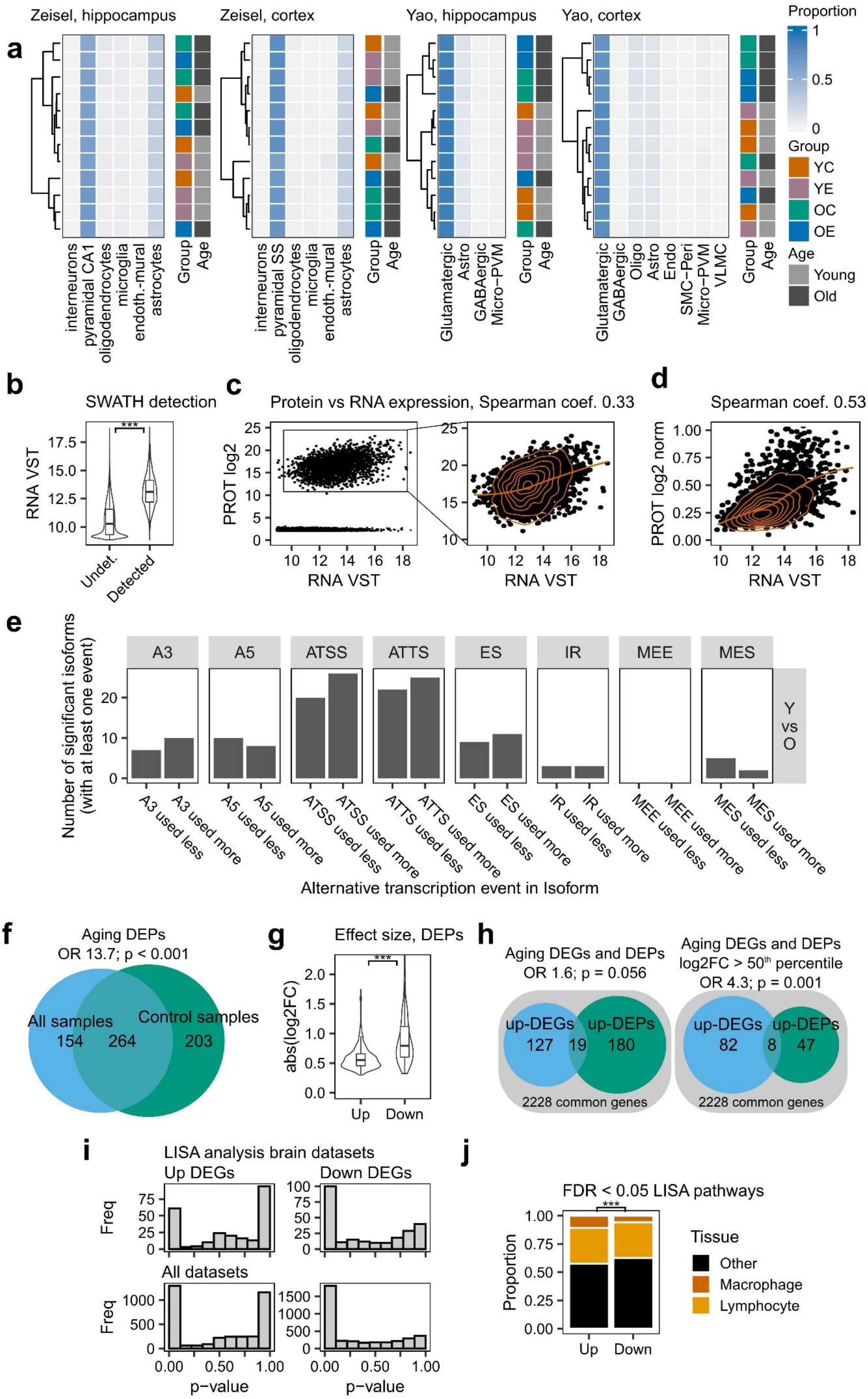
a
Zeisel, hippocampus
Zeisel, cortex
Yao, hippocampus
Yao, cortex
interneurons
pyramidal CA1
oligodendrocytes
microglia
endoth.-mural
astrocytes
pyramidal SS
Glutamatergic
Astro
GABAergic
Micro−PVM
Oligo
Endo
SMC−Peri
VLMC
Group
Age
Proportion
1
0.5
0
YC
YE
OC
OE
Young
Old
b
SWATH detection
RNA VST
17.5
15.0
12.5
10.0
***
Undet.
Detected
c
Protein vs RNA expression, Spearman coef. 0.33
PROT log2
RNA VST
d
Spearman coef. 0.53
PROT log2 norm
RNA VST
e
Number of significant isoforms (with at least one event)
A3
A5
ATSS
ATTS
ES
IR
MEE
MES
Y vs O
A3 used less
A3 used more
A5 used less
A5 used more
ATSS used less
ATSS used more
ATTS used less
ATTS used more
ES used less
ES used more
IR used less
IR used more
MEE used less
MEE used more
MES used less
MES used more
Alternative transcription event in Isoform
f
Aging DEPs
OR 13.7; p < 0.001
All samples
154
264
Control samples
203
g
Effect size, DEPs
abs(log2FC)
***
Up
Down
h
Aging DEGs and DEPs
OR 1.6; p = 0.056
up-DEGs
127
19
up-DEPs
180
2228 common genes
Aging DEGs and DEPs
log2FC > 50th percentile
OR 4.3; p = 0.001
up-DEGs
82
8
up-DEPs
47
2228 common genes
i
LISA analysis brain datasets
Up DEGs
Down DEGs
Freq
All datasets
p−value
j
FDR < 0.05 LISA pathways
Proportion
***
Up
Down
Tissue
Other
Macrophage
Lymphocyte

FIGURE S4

Fig. S4. The interplay between the transcriptomic and proteomic alterations of aging in the dorsal hippocampus. a, Heatmaps describing the estimated proportions of brain cell types inferred in the bulk RNA samples using the single-cell hippocampus and cortex datasets from Zeisel et al. (2015) and the single-cell hippocampus and cortex datasets from Yao et al. (2021). **b**, Violin plots showing the distribution of RNA expression levels (VST units) for RNA-seq genes which were detected or undetected in SWATH-MS (***p < 0.001 for Wilcoxon rank sum test). **c**, Scatter plots describing the quantitative relationship between SWATH-MS protein expression levels and RNA-seq gene expression levels for all RNA-seq detected genes (left) and genes with paired RNA-seq and SWATH-MS quantification (right). A LOESS curve is shown over the top of the data points. **d**, Scatter plot indicating the quantitative relationship between SWATH-MS protein expression levels, normalized by protein half-lives, and RNA-seq gene expression levels for genes with paired SWATH-MS and RNA-seq quantification. A LOESS curve is shown over the top of the data points. **e**, Bar plots describing the numbers of different types of alternative splicing events detected in isoforms with significant differential usage (FDR < 0.05, dIF >= 0.1, two-sided DEXseq tests) with aging in the RNA-seq data (A3, A5: alternative 3' acceptor or 5' donor sites; ATSS, ATTS: alternative transcription start or termination sites; ES: exon skipping; IR: intron retention; MES: multiple exon skipping; MEE: mutually exclusive exons). **f**, Venn diagram showing the overlap between aging DEPs (FDR < 0.05, two-sided moderated t-tests) detected using either all samples or only control samples. **g**, Violin plots indicating the distributions of absolute log2(fold change) for up- and down-regulated DEPs with aging (***p < 0.001 for Wilcoxon rank sum test). **h**, Venn diagrams describing the intersections between differentially expressed genes (DEGs) and proteins (DEPs) which displayed up-regulation in the aging comparison and had paired measurements for RNA and protein expression. The left plots show all significant genes (FDR < 0.05, two-sided Wald or moderated t-tests for RNA-seq and SWATH-MS data, respectively) while the right plots show significant genes with a log2(fold change) higher than the 50th percentile. **i**, Histograms showing the p-value distributions of the LISA enrichment analysis of regulators of aging-DEGs whereby downregulated genes show enrichment in more significant regulators across brain or all datasets. **j**, Bar plots comparing the proportion of LISA significant regulators (FDR < 0.05) belonging to macrophage-like datasets for aging up- and down-DEGs. (***p < 0.001 for Fisher's exact test).

FIGURE S5

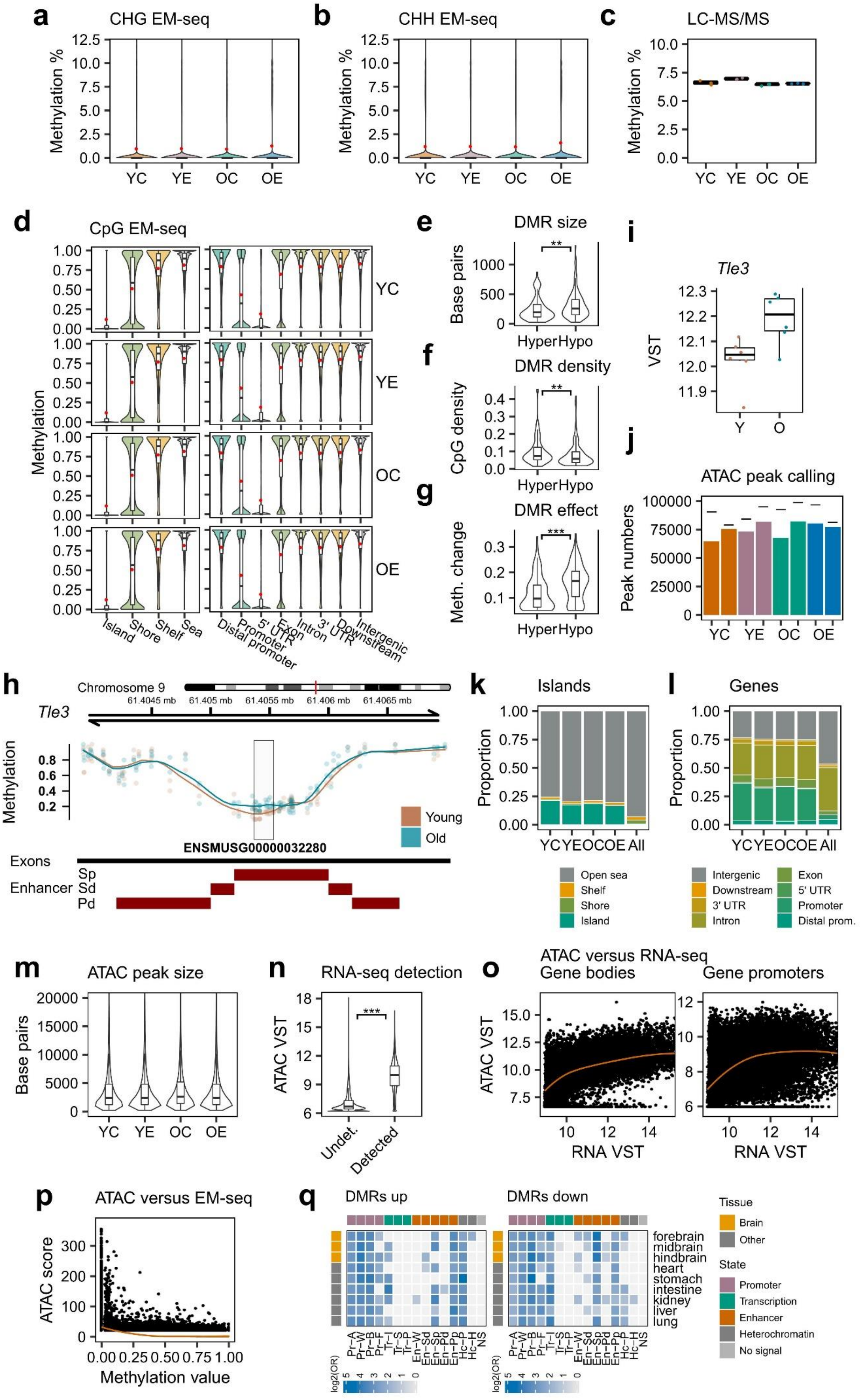

a
CHG EM-seq
Methylation %
YC YE OC OE
b
CHH EM-seq
c
LC-MS/MS
d
CpG EM-seq
Methylation
Island Shore Shelf Sea
Distal promoter Promoter 5' UTR Exon Intron 3' UTR Downstream Intergenic
YC
YE
OC
OE
e
DMR size
Base pairs
Hyper Hypo
f
DMR density
CpG density
g
DMR effect
Meth. change
i
Tle3
VST
Y O
j
ATAC peak calling
Peak numbers
h
Chromosome 9
Tle3
Methylation
ENSMUSG00000032280
Young
Old
Exons
Enhancer Sp Sd Pd
k
Islands
Proportion
YC YE OC OE All
Open sea
Shelf
Shore
Island
l
Genes
Intergenic
Downstream
3' UTR
Intron
Exon
5' UTR
Promoter
Distal prom.
m
ATAC peak size
Base pairs
n
RNA-seq detection
ATAC VST
Undet. Detected
o
ATAC versus RNA-seq
Gene bodies
Gene promoters
RNA VST
p
ATAC versus EM-seq
ATAC score
Methylation value
q
DMRs up
DMRs down
forebrain
midbrain
hindbrain
heart
stomach
intestine
kidney
liver
lung
log2(OR)
Tissue
Brain
Other
State
Promoter
Transcription
Enhancer
Heterochromatin
No signal

FIGURE S5

Fig. S5. DNA methylation and chromatin accessibility alterations during aging in the dorsal hippocampus. a-b, Violin plots showing the distribution of EM-seq methylation measurements of cytosines belonging to CHG (**a**) or CHH (**b**) genomic contexts across groups. Plots are based on a 1 M sample of Cs. Axes are cropped at 12.5 % methylation. **c**, Boxplots indicating the global methylation levels measured by LC-MS/MS in an independent set of samples. **d**, Violin plots describing the distributions of EM-seq methylation measurements of CpG sites across different genomic elements, segregated by group. **e-g**, Violin plots showing the distribution of size (**e**), density in CpG sites (**f**) and effect size (**g**) of aging-associated DMRs (**$p < 0.01$, ***$p < 0.001$ for Wilcoxon rank sum tests). **h**, Genomic plot showing the DNA methylation profiling values in young and old subjects of a region containing an aging-DMR (grey box) associated with the *Tle3* gene. Below, the tracks indicate the presence of gene exons and ENCODE murine forebrain P0 enhancer elements (Sp, Sd or Pd). **i**, Boxplot indicating the RNA-seq expression measurements (VST units) for the *Tle3* gene across young and old subjects. **j**, Bar plots describing the numbers of called ATAC-seq peaks (FDR < 0.05, epic2 peak calling test) across groups and replicates. The horizontal lines indicate the relative library size for each sample. **k-l,** Bar plots showing the proportion of ATAC-seq peaks (collapsed by replicates) mapped to CpG island (**k**) or gene (**l**) locations, as compared to the distribution of the whole genome (200 bp bins). **m**, Violin plots indicating the distribution of size in base pairs of ATAC-seq peaks across groups (collapsed by replicates). **n**, Violin plots showing the distribution of ATAC accessibility levels (VST units) in the body of genes which were detected or undetected in RNA-seq (***$p < 0.001$ for Wilcoxon rank sum test). **o**, Scatter plots describing the quantitative correlation between ATAC accessibility levels at gene bodies (left) and gene promoters (right) with RNA-seq gene expression levels for all RNA-seq detected genes. A LOESS curve is shown over the top of the data points. **p**, Scatter plot showing the quantitative correlation between ATAC accessibility levels at CpG sites with EM-seq DNA methylation levels for a sample of 100,000 profiled CpG sites. A LOESS curve is shown over the top of the data points. **q**, Heatmaps indicating the significant (FDR < 0.05, one-sided Fisher's exact tests) LOLA enrichments in log2(odds ratio) of chromatin states associated with aging-DMRs defined with FDR < 0.05 (two-sided metilene analysis tests). The code for the ENCODE chromatin states shown is: Promoter, Active (Pr-A), Weak (Pr-W), Bivalent (Pr-B) and Flanking (Pr-F); Enhancer, Strong TSS-distal (En-Sd), Strong TSS-proximal (En-Sp), Weak (En-W), Poised TSS-distal (En-Pd) and Poised TSS-proximal (En-Pp); Transcription, Strong (Tr-S), Permissive (Tr-P) and Initiation (Tr-I); Heterochromatin, Polycomb-associated (Hc-P) and H3K9me3-associated (Hc-H); No significant signal (NS).

FIGURE S6

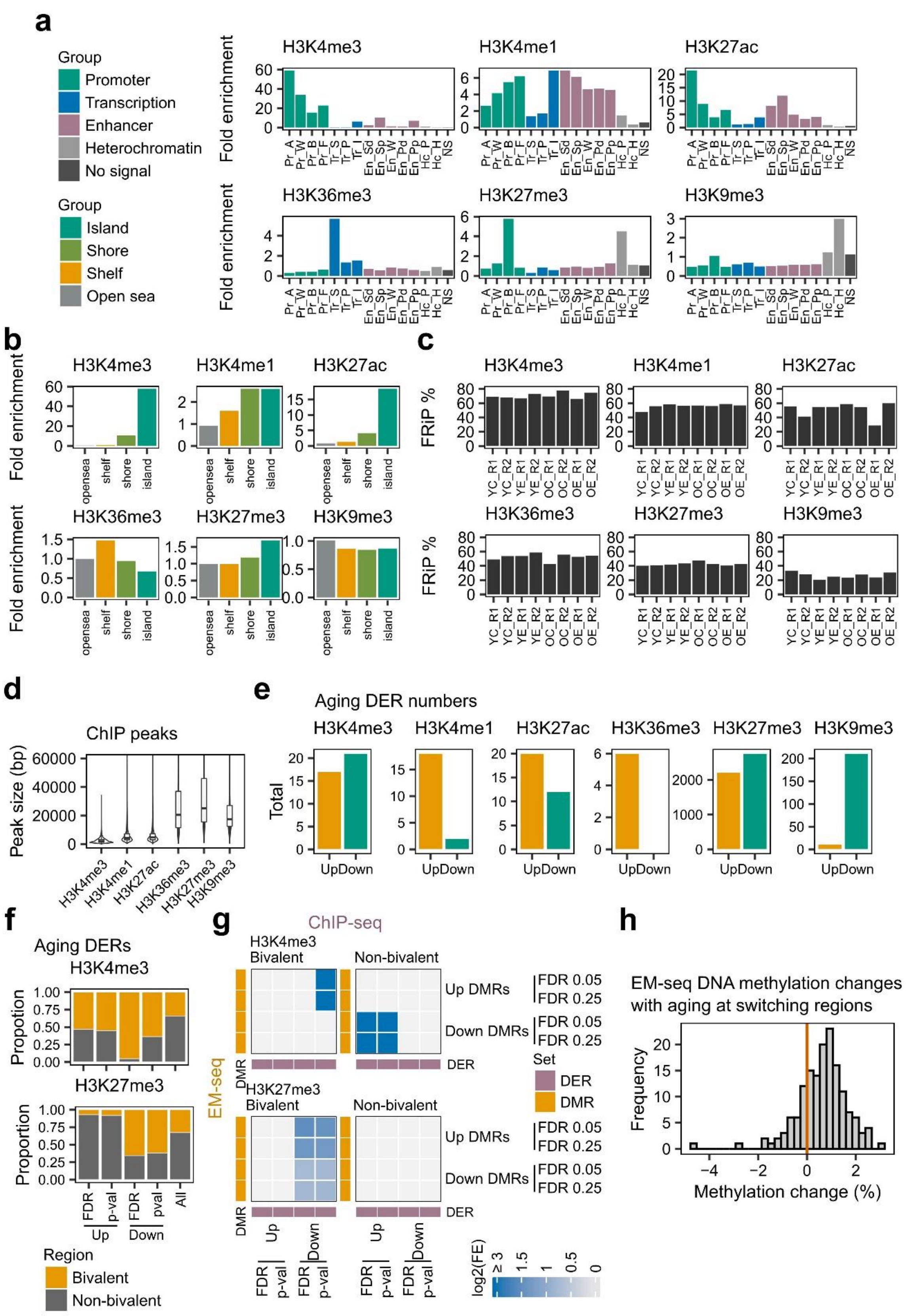

a
Group
Promoter
Transcription
Enhancer
Heterochromatin
No signal
Group
Island
Shore
Shelf
Open sea
Fold enrichment
H3K4me3
H3K4me1
H3K27ac
H3K36me3
H3K27me3
H3K9me3
b
c
FRiP %
d
ChIP peaks
Peak size (bp)
e
Aging DER numbers
Total
UpDown
f
Aging DERs
Propotion
Proportion
Region
Bivalent
Non-bivalent
g
ChIP-seq
EM-seq
Bivalent
Non-bivalent
Up DMRs
Down DMRs
FDR 0.05
FDR 0.25
Set
DER
DMR
log2(FE)
h
EM-seq DNA methylation changes with aging at switching regions
Frequency
Methylation change (%)

FIGURE S6

Fig. S6. The chromatin landscape of the dorsal hippocampus during aging. a-b, Bar plots indicating the enrichment of the different profiled ChIP-seq histone marks in ENCODE murine forebrain P0 chromatin states (**a**) and CpG island locations (**b**). The enrichment is measured as the percentage of reads mapped to a certain genomic element compared to the percentage of the Input sample. All samples and groups are averaged. The code for the ENCODE chromatin states shown is: Promoter, Active (Pr-A), Weak (Pr-W), Bivalent (Pr-B) and Flanking (Pr-F); Enhancer, Strong TSS-distal (En-Sd), Strong TSS-proximal (En-Sp), Weak (En-W), Poised TSS-distal (En-Pd) and Poised TSS-proximal (En-Pp); Transcription, Strong (Tr-S), Permissive (Tr-P) and Initiation (Tr-I); Heterochromatin, Polycomb-associated (Hc-P) and H3K9me3-associated (Hc-H); No significant signal (NS). **c**, Bar plots showing the FRiP scores of the different samples for each histone mark using their respective consensus peak set. **d**, Violin plots describing the distribution of size in base pairs of the consensus ChIP-seq peak sets across each histone mark. **e**, Bar plots showing the numbers of DERs (FDR < 0.05, two-sided Wald tests) with increased or decreased levels associated with aging across each histone modification. **f**, Bar plots indicating the proportion of aging up- or down-DERs (filtered either by FDR < 0.05 or p-value < 0.05, two-sided Wald tests) which overlap with bivalent chromatin domains for the H3K4me3 and the H3K27me3 modifications. The last bar plot indicates the proportion of all of the consensus peaks mapped to bivalent domains. **g**, Heatmaps describing the log2-fold enrichment of significant intersections (FDR < 0.05 within each set for one-sided permutation regioneR tests) between H3K4me3 and H3K27me3 ChIP-seq aging DERs (filtered either at FDR < 0.05 or p-value < 0.05, two-sided Wald tests) and EM-seq DMRs (filtered either at FDR < 0.05 or < 0.25, two-sided metilene analysis tests). The ChIP-seq DERs are separated into those that overlap bivalent domains and those that do not. **h**, Histogram showing the distribution of EM-seq DNA methylation differences with aging at heterochromatin switching regions, defined as the intersection between aging H3K27me3 up-DERs (FDR < 0.05, two-sided Wald tests) and aging H3K9me3 down-DERs (FDR < 0.05, two-sided Wald tests). The differences are computed by taking the average of the methylation values of the EM-seq profiled CpG sites at each region and calculating the difference between old and young samples (old - young).

FIGURE S7

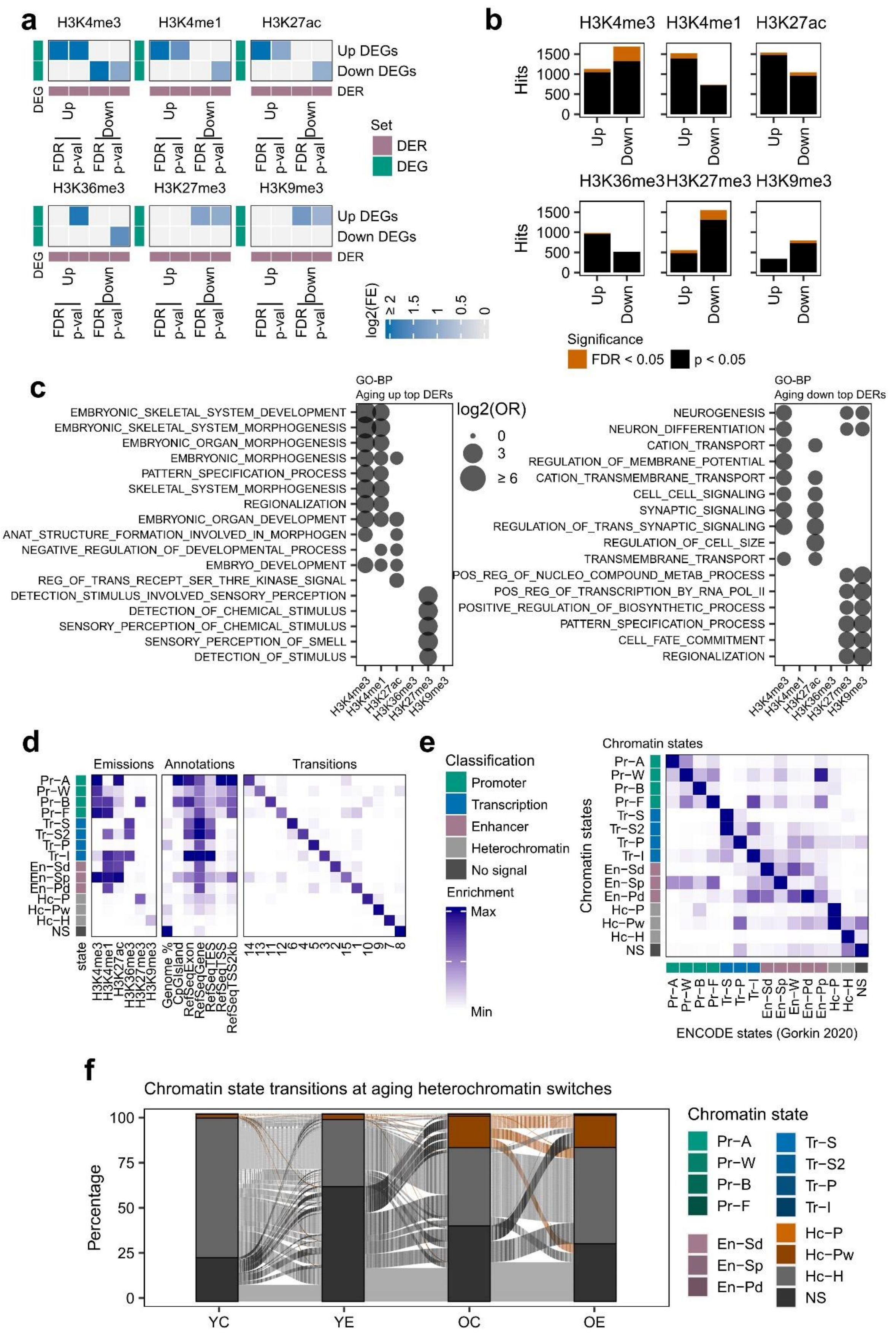
a
H3K4me3
H3K4me1
H3K27ac
Up DEGs
Down DEGs
DER
DEG
Up
Down
FDR
p-val
Set
DER
DEG
H3K36me3
H3K27me3
H3K9me3
log2(FE)
≥ 2
1.5
1
0.5
0
b
Hits
1500
1000
500
0
Significance
FDR < 0.05
p < 0.05
c
GO-BP
Aging up top DERs
EMBRYONIC_SKELETAL_SYSTEM_DEVELOPMENT
EMBRYONIC_SKELETAL_SYSTEM_MORPHOGENESIS
EMBRYONIC_ORGAN_MORPHOGENESIS
EMBRYONIC_MORPHOGENESIS
PATTERN_SPECIFICATION_PROCESS
SKELETAL_SYSTEM_MORPHOGENESIS
REGIONALIZATION
EMBRYONIC_ORGAN_DEVELOPMENT
ANAT_STRUCTURE_FORMATION_INVOLVED_IN_MORPHOGEN
NEGATIVE_REGULATION_OF_DEVELOPMENTAL_PROCESS
EMBRYO_DEVELOPMENT
REG_OF_TRANS_RECEPT_SER_THRE_KINASE_SIGNAL
DETECTION_STIMULUS_INVOLVED_SENSORY_PERCEPTION
DETECTION_OF_CHEMICAL_STIMULUS
SENSORY_PERCEPTION_OF_CHEMICAL_STIMULUS
SENSORY_PERCEPTION_OF_SMELL
DETECTION_OF_STIMULUS
log2(OR)
0
3
≥ 6
GO-BP
Aging down top DERs
NEUROGENESIS
NEURON_DIFFERENTIATION
CATION_TRANSPORT
REGULATION_OF_MEMBRANE_POTENTIAL
CATION_TRANSMEMBRANE_TRANSPORT
CELL_CELL_SIGNALING
SYNAPTIC_SIGNALING
REGULATION_OF_TRANS_SYNAPTIC_SIGNALING
REGULATION_OF_CELL_SIZE
TRANSMEMBRANE_TRANSPORT
POS_REG_OF_NUCLEO_COMPOUND_METAB_PROCESS
POS_REG_OF_TRANSCRIPTION_BY_RNA_POL_II
POSITIVE_REGULATION_OF_BIOSYNTHETIC_PROCESS
PATTERN_SPECIFICATION_PROCESS
CELL_FATE_COMMITMENT
REGIONALIZATION
H3K4me3
H3K4me1
H3K27ac
H3K36me3
H3K27me3
H3K9me3
d
Emissions
Annotations
Transitions
Pr−A
Pr−W
Pr−B
Pr−F
Tr−S
Tr−S2
Tr−P
Tr−I
En−Sd
En−Sp
En−Pd
Hc−P
Hc−Pw
Hc−H
NS
state
Genome %
CpGIsland
RefSeqExon
RefSeqGene
RefSeqTES
RefSeqTSS
RefSeqTSS2kb
e
Classification
Promoter
Transcription
Enhancer
Heterochromatin
No signal
Enrichment
Max
Min
Chromatin states
En−W
En−Pp
ENCODE states (Gorkin 2020)
f
Chromatin state transitions at aging heterochromatin switches
Percentage
100
75
50
25
0
YC
YE
OC
OE
Chromatin state

FIGURE S7

Fig. S7. Functional associations between chromatin alterations during aging in the dorsal hippocampus. a, Heatmaps describing the log2-fold enrichment of significant intersections (FDR < 0.05 within each set for one-sided permutation regioneR tests) between ChIP-seq aging DERs (filtered either at FDR < 0.05 or p-value < 0.05, two-sided Wald tests) and RNA-seq aging DEGs (FDR < 0.05, two-sided Wald tests). **b**, Bar plots showing the numbers of enriched pathways (p < 0.05 or FDR < 0.05, one-sided Wallenius tests) detected in the gene set enrichment analyses across various databases (Gene Ontology, Chemical and Genetic Perturbation, Reactome, WikiPathways, ImmuneSigDB, C8 cell type signatures) for aging up- and downregulated top DERs (unadjusted p < 0.05, two-sided Wald tests) across each histone mark. **c**, Bubble plots indicating the top 5 pathways found enriched (FDR < 0.05, one-sided Wallenius tests) across the Gene Ontology Biological Process database for genes associated with aging top up- and down-DERs (unadjusted p < 0.05, two-sided Wald tests) across each histone mark. The size of the bubbles indicates the log2(odds ratio) of enrichment. **d**, Heatmaps describing the emission probabilities for the histone modifications at each learned chromatin state (left), the enrichments of each state at various genomic locations (centre; normalized to [0-1] by columns) and the transition probabilities between each state. **e**, Heatmap showing the enrichments between the learned chromatin states and ENCODE3 mouse P0 forebrain states (normalized to [0-1] by rows). **f**, Alluvial diagram showing the chromatin state annotations (200 bp genomic bins) at the previously characterized regions of aging-associated heterochromatin switching. The lines connecting the bar plots show the transitions in chromatin state annotations between the experimental groups. The code for the chromatin states learnt in this study is: Promoter, Active (Pr-A), Weak (Pr-W), Bivalent (Pr-B) and Flanking (Pr-F); Transcription, Strong (Tr-S), Strong 2 (Tr-S2), Permissive (Tr-P) and Initiation (Tr-I); Enhancer, Strong TSS-distal (En-Sd), Strong TSS-proximal (En-Sp) and Poised TSS-distal (En-Pd); Heterochromatin, Polycomb-associated (Hc-P), Polycomb-associated weak (Hc-Pw) and H3K9me3-associated (Hc-H); No significant signal (NS). The code for the ENCODE chromatin states shown is: Promoter, Active (Pr-A), Weak (Pr-W), Bivalent (Pr-B) and Flanking (Pr-F); Enhancer, Strong TSS-distal (En-Sd), Strong TSS-proximal (En-Sp), Weak (En-W), Poised TSS-distal (En-Pd) and Poised TSS-proximal (En-Pp); Transcription, Strong (Tr-S), Permissive (Tr-P) and Initiation (Tr-I); Heterochromatin, Polycomb-associated (Hc-P) and H3K9me3-associated (Hc-H); No significant signal (NS).

FIGURE S8

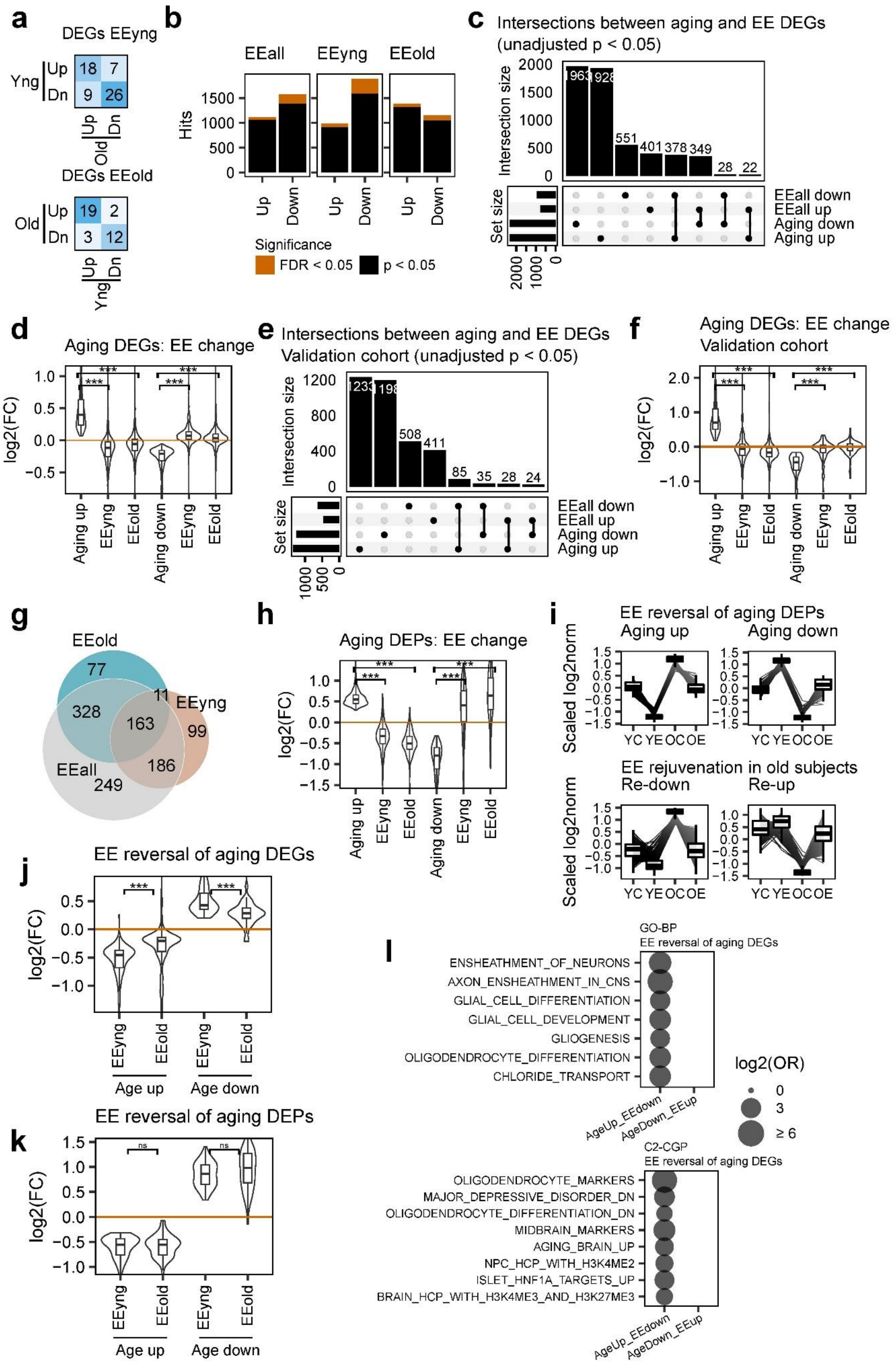

a
DEGs EEyng
DEGs EEold
b
EEall
EEyng
EEold
Hits
Significance
FDR < 0.05
p < 0.05
c
Intersections between aging and EE DEGs
(unadjusted p < 0.05)
Intersection size
Set size
EEall down
EEall up
Aging down
Aging up
d
Aging DEGs: EE change
log2(FC)
e
Intersections between aging and EE DEGs
Validation cohort (unadjusted p < 0.05)
f
Aging DEGs: EE change
Validation cohort
g
EEold
EEyng
EEall
h
Aging DEPs: EE change
i
EE reversal of aging DEPs
Aging up
Aging down
EE rejuvenation in old subjects
Re-down
Re-up
Scaled log2norm
j
EE reversal of aging DEGs
Age up
Age down
k
EE reversal of aging DEPs
l
GO-BP
EE reversal of aging DEGs
ENSHEATHMENT_OF_NEURONS
AXON_ENSHEATHMENT_IN_CNS
GLIAL_CELL_DIFFERENTIATION
GLIAL_CELL_DEVELOPMENT
GLIOGENESIS
OLIGODENDROCYTE_DIFFERENTIATION
CHLORIDE_TRANSPORT
AgeUp_EEdown
AgeDown_EEup
log2(OR)
C2-CGP
EE reversal of aging DEGs
OLIGODENDROCYTE_MARKERS
MAJOR_DEPRESSIVE_DISORDER_DN
OLIGODENDROCYTE_DIFFERENTIATION_DN
MIDBRAIN_MARKERS
AGING_BRAIN_UP
NPC_HCP_WITH_H3K4ME2
ISLET_HNF1A_TARGETS_UP
BRAIN_HCP_WITH_H3K4ME3_AND_H3K27ME3

FIGURE S8

Fig. S8. Functional interactions between aging and environmental enrichment in the dorsal hippocampus. a, Heatmaps displaying the numbers and direction of RNA-seq EE-DEGs in young (left) and old (right) samples, compared to the direction of change of the same genes in the old (left) or young (right) groups. **b**, Bar plots showing the number of enriched pathways ($p < 0.05$ or FDR < 0.05, one-sided Wallenius tests) detected in the gene set enrichment analyses across various databases (Gene Ontology, Chemical and Genetic Perturbation, Reactome, WikiPathways, ImmuneSigDB, C8 cell type signatures) for EE-associated up- and down-regulated top DEGs (unadjusted $p < 0.05$, two-sided Wald tests) in all, young or old samples. **c**, UpSet plot describing the intersections between aging and EEall top DEGs (unadjusted $p < 0.05$, two-sided Wald tests). **d**, Violin plots showing the log2(fold change) values for the aging up- and down-DEGs (FDR < 0.05, two-sided Wald tests) and also, for the same sets of genes, the fold change values with EE in young and old samples (***$p < 0.001$ for Wilcoxon rank sum tests). **e**, UpSet plot indicating the intersections between aging and EEall top DEGs (unadjusted $p < 0.05$, two-sided Wald tests) for the external validation cohort. **f**, Violin plots showing the log2(fold change) values for the aging up- and down-DEGs (FDR < 0.05, two-sided Wald tests) for the external validation cohort and also, for the same sets of genes, the fold change values with EE in young and old samples (***$p < 0.001$ for Wilcoxon rank sum tests). **g**, Venn diagram describing the intersections between EE-associated DEPs for young, old and all samples. **h**, Violin plots showing the log2(fold change) values for the aging up- and down-DEPs (FDR < 0.05, two-sided moderated t-tests) and also, for the same sets of genes, the fold change values with EE in young and old samples (***$p < 0.001$ for Wilcoxon rank sum tests). **i**, Line plots showing the protein expression values (scaled log2-normalized units) of curated genes which show opposing aging and EE-reversal changes (top) or rejuvenation specifically in old subjects (bottom). **j**, Violin plots showing the log2(fold change) values in young or old samples for the aging up-EE down, or the aging down-EE up reversal genes (***$p < 0.001$ for Wilcoxon rank sum tests). **k**, Violin plots showing the log2(fold change) values in young or old samples for the aging up-EE down, or the aging down-EE up reversal proteins ($^{ns}p > 0.05$ for Wilcoxon rank sum tests). **l**, Bubble plots describing the pathways found enriched (FDR < 0.05, one-sided Wallenius tests) across the Gene Ontology Biological Process and C2 CGP (Chemical and Genetic Perturbation) databases for curated RNA-seq EE-reversal (in all samples) genes. The size of the bubbles indicates the log2(odds ratio) of enrichment.

FIGURE S9

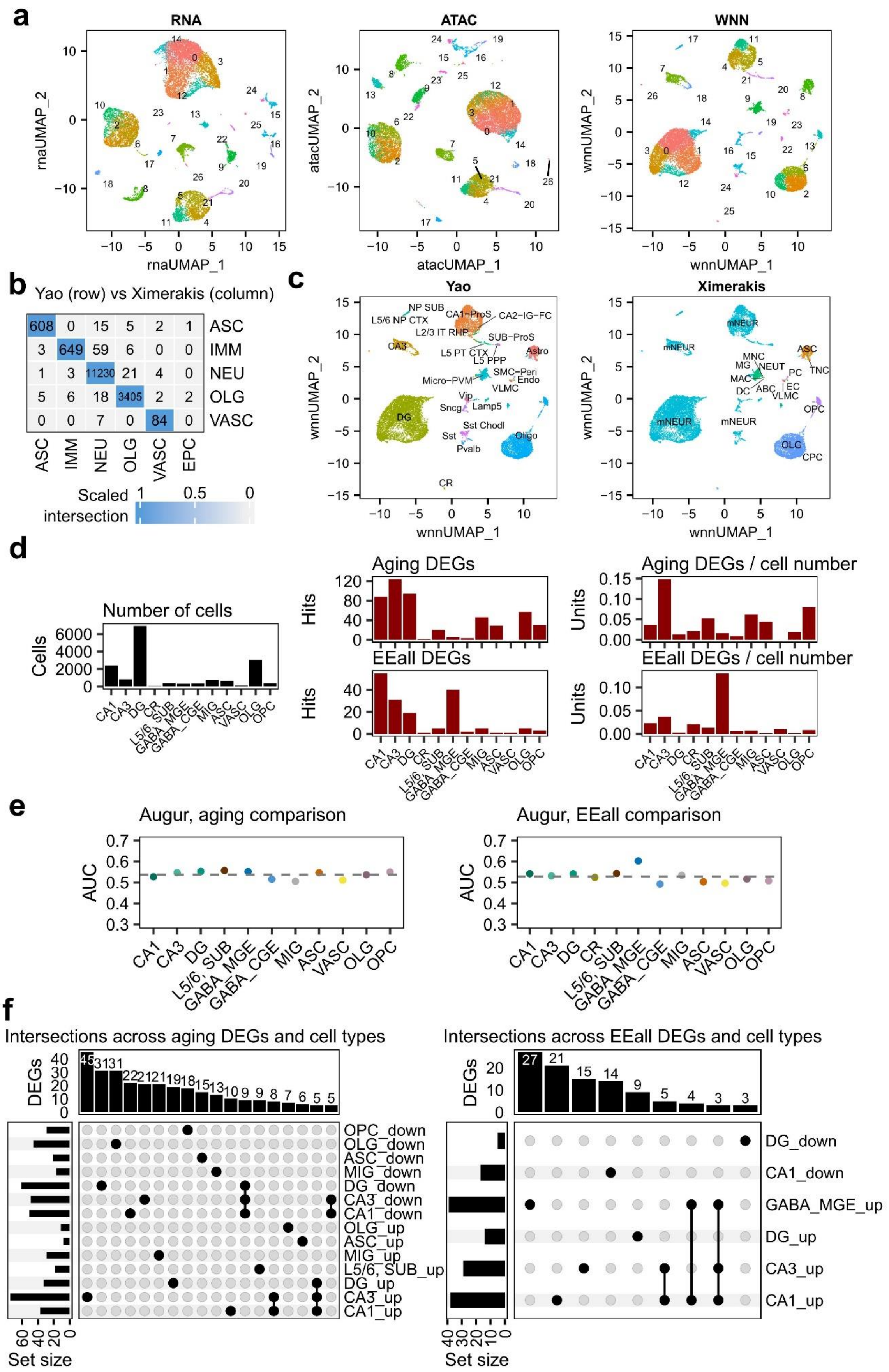
a
RNA
ATAC
WNN
rnaUMAP_1
rnaUMAP_2
atacUMAP_1
atacUMAP_2
wnnUMAP_1
wnnUMAP_2
b
Yao (row) vs Ximerakis (column)
ASC
IMM
NEU
OLG
VASC
EPC
Scaled intersection
c
Yao
Ximerakis
d
Number of cells
Cells
Aging DEGs
Hits
Aging DEGs / cell number
Units
EEall DEGs
EEall DEGs / cell number
e
Augur, aging comparison
Augur, EEall comparison
AUC
f
Intersections across aging DEGs and cell types
Intersections across EEall DEGs and cell types
DEGs
Set size

FIGURE S9

Fig. S9. The single cell dynamics of aging and environmental enrichment in the dorsal hippocampus. a, Dimensional reduction plots showing the distribution of cells, labelled by WNN clusters, for the scRNA-seq reduction (integrated PCA, left), the scATAC-seq reduction (integrated LSI, middle) and the final multimodal WNN UMAP reduction (right). **b**, Heatmap describing the number of matching annotations, across major cell types, obtained by using either the Yao or the Ximerakis dataset for cell type annotation (NEU: neuron; OLG: oligodendrocyte or progenitor; ASC: astrocyte; IMM: microglia or perivascular macrophage; VASC: vascular cell, endothelial cell, smooth muscle cell or pericyte; EPC: ependymocytes). **c**, Dimensional reduction plots indicating the cell type annotations assigned when using the Yao (left) or Ximerakis (right) datasets. **d**, Bar plots indicating, from left to right, the number of cells analysed per cell type, the number of aging or EEall DEGs detected per cell type (adj. $p < 0.05$, $logFC > 0.25$, using all samples in the four groups to compare aging or EE, two-sided likelihood ratio tests), and the number of DEGs divided by the total number of cells in each population. **e**, Scatter plot showing the "area under the receiver operating characteristic curve" (AUC) values computed by Augur for the aging or EE comparison across cell types. Higher AUC values indicate a stronger effect of the comparison in terms of numbers of DEGs and effect size. **f**, UpSet plots describing the intersections between aging and EEall DEGs across cell types. The aging plot shows sets containing >= 5 DEGs, while the EE plot shows sets with >= 3 DEGs. The code used for the Yao cell type annotations is: DG (Neuron, glutamatergic, dentate gyrus), CA1-ProS (Neuron, glutamatergic, CA1, Prosubiculum), CA2-IG-FC (Neuron, glutamatergic CA2 IG or FC regions), CA3 (Neuron, glutamatergic, CA3), SUB-ProS (Neuron, Subiculum, Prosubiculum), NP SUB (Neuron, near-projecting, subiculum), NP PPP (Neuron, near-projecting, subiculum related), L5 PT CTX, L5 PPP and L5/6 NP CTX (Neuron, glutamatergic, Cortical layer 5 or 6, pyramidal tract, cortex (CTX) or subiculum (PPP) related), L6 CT, L6b CTX and L6b/CT ENT (Neuron, glutamatergic, Cortical layer 6 or 6b pyramidal corticothalamic, cortex or ENT related), L2/3 IT RHP (Neuron, glutamatergic, intratelencephalic related, retrohippocampal region), Sst and Pvalb (Neuron, GABAergic, medial ganglionic eminence (MGE) origin, Sst or Pvalb marker), Vip, Sncg and Lamp5 (Neuron, GABAergic, caudal ganglionic eminence (CGE) origin, Vip, Sncg or Lamp marker), CR (Neuron, Cajal-Retzius), Oligo (Oligodendrocyte), Astro (Astrocyte), Endo (Endothelial cell), SMC-Peri (Smooth muscle/pericyte), VLMC (vascular/leptomeningeal cell), PVM (microglia/perivascular macrophage). The code used for the Ximerakis cell type annotations is: mNEUR (Neuron), OLG (Oligodendrocyte), OPC (Oligodendrocyte progenitor), ASC (Astrocyte), TNC (tanycyte), CPC (choroid plexus epithelial cell), VLMC (vascular/leptomeningeal cell), PC (pericyte), ABC (arachnoid barrier cell), DC (dendritic cell), MAC (macrophage), MNC (monocyte), NEUT (neutrophil), EC (endothelial cell), MG (microglia).

FIGURE S10

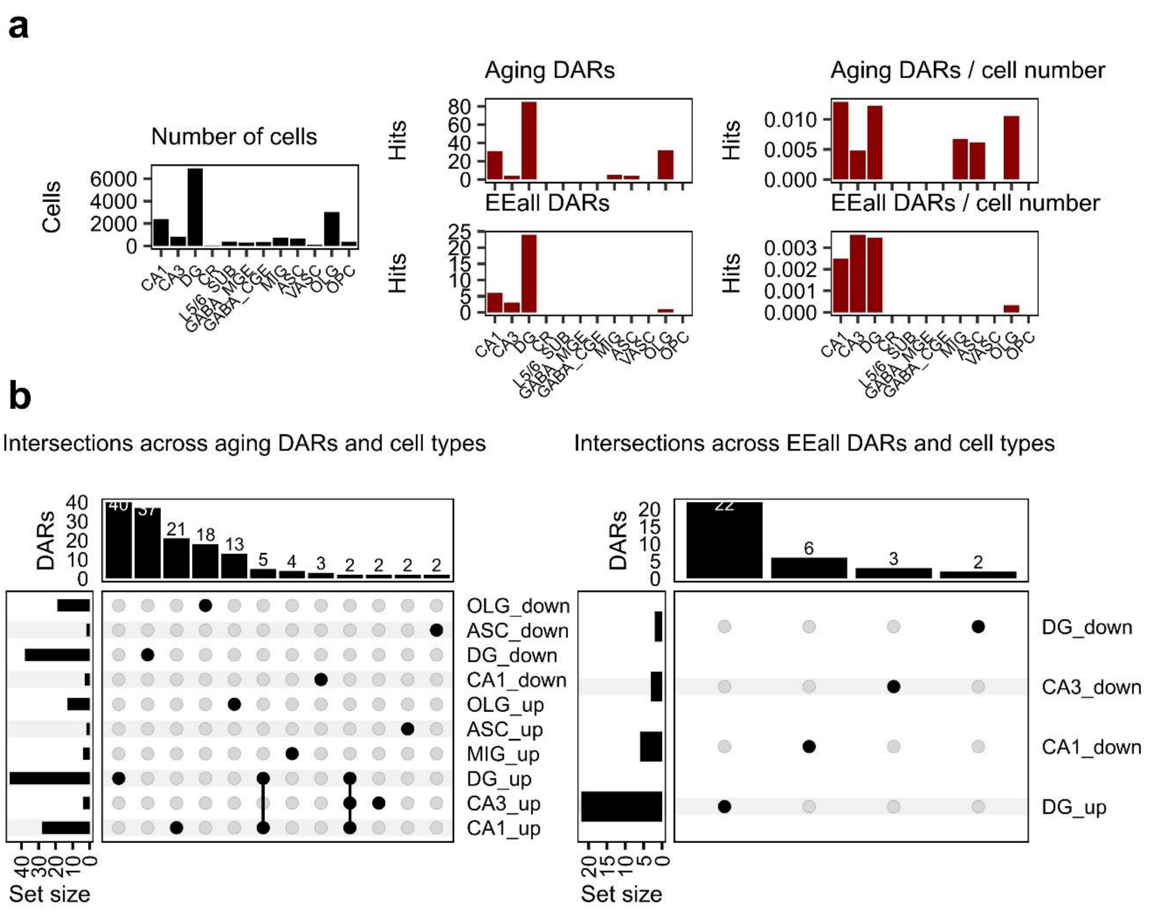
a
Number of cells
Cells
Aging DARs
Hits
Aging DARs / cell number
EEall DARs
EEall DARs / cell number
CA1
CA3
DG
CR
SUB
L5/6
GABA_MGE
GABA_CGE
MIG
ASC
VASC
OLG
OPC
b
Intersections across aging DARs and cell types
Intersections across EEall DARs and cell types
DARs
OLG_down
ASC_down
DG_down
CA1_down
OLG_up
ASC_up
MIG_up
DG_up
CA3_up
CA1_up
CA3_down
Set size

FIGURE S10

Fig. S10. Chromatin alterations with aging and environmental enrichment at the single-cell level in the dorsal hippocampus. a, Bar plots indicating, from left to right, the number of cells analysed per cell type, the number of aging or EEall DARs detected per cell type (adj. $p < 0.05$, $logFC > 0.25$, using all samples in the four groups to compare aging or EE, two-sided likelihood ratio tests) and the number of DARs divided by the total number of cells in each population. **b**, UpSet plots describing the intersections between aging and EEall DARs across cell types. The plots show sets containing >= 2 DARs.
