# Supplementary material for: A multiomic atlas of the aging hippocampus reveals molecular changes in response to environmental enrichment
Source: Nat Commun. 2024 Jul 16;15:5829. doi: 10.1038/s41467-024-49608-z (PMC11252340; doi:10.1038/s41467-024-49608-z)
Supplement: Supplementary file 3 — Description of Additional Supplementary Files [file 41467_2024_49608_MOESM3_ESM.pdf]

DESCRIPTION OF ADDITIONAL SUPPLEMENTARY FILES

Description of additional supplementary files for the manuscript "A MULTIOMIC ATLAS OF THE AGING HIPPOCAMPUS REVEALS MOLECULAR CHANGES IN RESPONSE TO ENVIRONMENTAL ENRICHMENT". It describes Supplementary Datasets 1 to 27.

SUPPLEMENTARY DATASETS

File Name: Supplementary Dataset 1.
Description: Description of the samples and omic experiments carried out in the study.

File Name: Supplementary Dataset 2.
Description: Differential gene expression results for aging transcriptomic comparisons. P-values correspond to two-sided Wald tests and are adjusted for multiple testing by FDR.

File Name: Supplementary Dataset 3.
Description: Gene pathway enrichment results for aging DEGs. The databases interrogated are: Gene Ontology, WikiPathways, Reactome, ImmuneSigDB and cell type signatures (C8) and chemical and genetic perturbation pathways (CGP) from MSigDB. P-values correspond to one-sided Wallenius tests and are adjusted for multiple testing by FDR.

File Name: Supplementary Dataset 4.
Description: Differential splicing analyses results for aging transcriptomic comparisons. P-values correspond to two-sided DEXseq tests and are adjusted for multiple testing by FDR.

File Name: Supplementary Dataset 5.
Description: Differential protein expression results for aging proteomic comparisons. The last sheet lists common aging DEPs (proteins) and DEGs (genes) which change in the same direction ($FDR < 0.05$). P-values correspond to two-sided moderated t-tests and are adjusted for multiple testing by FDR.

File Name: Supplementary Dataset 6.
Description: Gene pathway enrichment results for aging DEPs. The first sheet includes results for the intersections between top pathways (unadjusted $p < 0.05$, one-sided Wallenius tests) detected as enriched for aging DEGs (RNA-seq, $FDR < 0.05$, two-sided Wald tests) and aging DEPs (SWATH-MS, $FDR < 0.05$, two-sided moderated t-tests). The rest of the sheets detail the pathway enrichment results for aging DEPs. The databases interrogated are: Gene Ontology, WikiPathways, Reactome, ImmuneSigDB and cell type signatures (C8) and chemical and genetic perturbation pathways (CGP) from MSigDB. P-values correspond to one-sided Wallenius tests and are adjusted for multiple testing by FDR.

File Name: Supplementary Dataset 7.
Description: LISA analysis results for epigenomic regulators enriched in aging DEGs. P-values correspond to LISA tests and are adjusted for multiple testing by FDR.

File Name: Supplementary Dataset 8.
Description: Differentially methylated regions for aging EM-seq comparisons. The second sheet includes a selection of differentially methylated regions which map to aging DEGs. P-values correspond to metilene analysis tests involving 2D KS (two-dimensional Kolmogorov-Smirnov) tests and MWU (Mann-Whitney U) tests. The p-values for the MWU-tests are adjusted for multiple testing by FDR.

File Name: Supplementary Dataset 9.
Description: Gene pathway enrichment results for genes associated with aging DMRs. The databases interrogated are: Gene Ontology, WikiPathways, Reactome, ImmuneSigDB and cell type signatures (C8) and chemical and genetic perturbation pathways (CGP) from MSigDB. P-values correspond to one-sided Wallenius tests and are adjusted for multiple testing by FDR.

File Name: Supplementary Dataset 10.
Description: Differentially accessible regions for aging ATAC-seq comparisons. The second sheet includes a selection of differentially accessible regions (FDR < 0.05, two-sided Wald tests) which map to aging DEGs. The third sheet includes a selection of differentially accessible regions (unadjusted p < 0.05, two-sided Wald tests) which map to aging DMRs (FDR < 0.05, metilene analysis tests).

File Name: Supplementary Dataset 11.
Description: LOLA enrichments in chromatin states for differentially accessible regions (filtered for FDR < 0.05 or unadjusted p < 0.05, one-sided Fisher's exact tests).

File Name: Supplementary Dataset 12.
Description: LOLA enrichments in chromatin states for differentially methylated regions (filtered for FDR < 0.05, one-sided Fisher's exact tests).

File Name: Supplementary Dataset 13.
Description: Differentially enriched regions for aging ChIP-seq comparisons. P-values correspond to two-sided Wald tests and are adjusted for multiple testing by FDR.

File Name: Supplementary Dataset 14.
Description: Heterochromatin switching regions, defined as the intersection of H3K9me3 aging down-DERs and H3K27me3 aging up-DERs.

File Name: Supplementary Dataset 15.
Description: Gene pathway enrichment results for genes associated with heterochromatin switching regions, defined as the intersection of H3K9me3 aging down-DERs and H3K27me3 aging up-DERs. Enrichments are shown for single genes mapping to these regions or for genes flanking up to 50 kb from the regions. The databases interrogated are: Gene Ontology, WikiPathways, Reactome, ImmuneSigDB and cell type signatures (C8) and chemical and genetic perturbation pathways (CGP) from MSigDB. P-values correspond to one-sided Wallenius tests and are adjusted for multiple testing by FDR.

File Name: Supplementary Dataset 16.
Description: Gene pathway enrichment results for genes associated with the top aging DERs (unadjusted p < 0.05, two-sided Wald tests) across each histone mark. The databases interrogated are: Gene Ontology, WikiPathways, Reactome, ImmuneSigDB and cell type signatures (C8) and chemical and genetic perturbation pathways (CGP) from MSigDB. P-values correspond to one-sided Wallenius tests and are adjusted for multiple testing by FDR.

File Name: Supplementary Dataset 17.
Description: Annotation of chromatin states from the histone modification data. The first sheet describes the assigned biological states. The second sheet indicates the emission probabilities for the histone modifications at each state. The third sheet shows the transition probabilities across the states. The rest of the sheets indicate the enrichment of states at different genomic

locations, TSS and TES neighbourhood and ENCODE3 chromatin state tracks for mouse postnatal P0 forebrain.

File Name: Supplementary Dataset 18.
Description: Differential gene expression results for environmental enrichment transcriptomic comparisons. P-values correspond to two-sided Wald tests and are adjusted for multiple testing by FDR.

File Name: Supplementary Dataset 19.
Description: Gene pathway enrichment results for the top EE-associated DEGs (unadjusted $p < 0.05$). The databases interrogated are: Gene Ontology, WikiPathways, Reactome, ImmuneSigDB and cell type signatures (C8) and chemical and genetic perturbation pathways (CGP) from MSigDB. P-values correspond to one-sided Wallenius tests and are adjusted for multiple testing by FDR.

File Name: Supplementary Dataset 20.
Description: Differential protein expression results for environmental enrichment proteomic comparisons. P-values correspond to two-sided moderated t-tests and are adjusted for multiple testing by FDR.

File Name: Supplementary Dataset 21.
Description: Selection of genes displaying EE-associated reversal or rejuvenation of aging gene expression alterations at the RNA or protein level. Rejuvenated genes are defined as stated in Methods. The last sheet includes a selection of EE-reversal genes with consistent measurements of RNA and protein alterations as described in Methods.

File Name: Supplementary Dataset 22.
Description: Gene pathway enrichment results for the curated RNA-seq aging-EE reversal genes. The databases interrogated are: Gene Ontology, WikiPathways, Reactome, ImmuneSigDB and cell type signatures (C8) and chemical and genetic perturbation pathways (CGP) from MSigDB. P-values correspond to one-sided Wallenius tests and are adjusted for multiple testing by FDR.

File Name: Supplementary Dataset 23.
Description: Differentially enriched regions for environmental enrichment ChIP-seq comparisons. P-values correspond to two-sided Wald tests and are adjusted for multiple testing by FDR.

File Name: Supplementary Dataset 24.
Description: Selection of regions displaying EE-associated rejuvenation of aging alterations in histone post-translational modifications. The rejuvenated regions are defined as stated in Methods. The last sheet includes a selection of rejuvenation DERs which overlap with previously defined rejuvenation DEGs.

File Name: Supplementary Dataset 25.
Description: Cell type annotation of the single-cell WNN clusters.

File Name: Supplementary Dataset 26.
Description: Differential gene expression results for aging and EE comparisons in the single cell data. Comparisons carried out across major cell types. P-values correspond to two-sided likelihood ratio tests and are adjusted for multiple testing by the Bonferroni method.

File Name: Supplementary Dataset 27.
Description: Differential accessibility results for aging and EE comparisons in the single-cell data. Comparisons carried out across major cell types. The results are filtered to show DARs with unadjusted $p < 0.05$. P-values correspond to two-sided likelihood ratio tests and are adjusted for multiple testing by the Bonferroni method.
