# Supplementary material for: A multiomic atlas of the aging hippocampus reveals molecular changes in response to environmental enrichment
Source: Nat Commun. 2024 Jul 16;15:5829. doi: 10.1038/s41467-024-49608-z (PMC11252340; doi:10.1038/s41467-024-49608-z)
Supplement: Supplementary file 31 — Reporting Summary [file 41467_2024_49608_MOESM31_ESM.pdf]

nature portfolio

Corresponding author(s): Mario F. Fraga, Agustín F. Fernández

Last updated by author(s): May 13, 2024

Reporting Summary

Nature Portfolio wishes to improve the reproducibility of the work that we publish. This form provides structure for consistency and transparency in reporting. For further information on Nature Portfolio policies, see our Editorial Policies and the Editorial Policy Checklist.

Statistics

For all statistical analyses, confirm that the following items are present in the figure legend, table legend, main text, or Methods section.

n/a	Confirmed	
☐	☒	The exact sample size (*n*) for each experimental group/condition, given as a discrete number and unit of measurement
☐	☒	A statement on whether measurements were taken from distinct samples or whether the same sample was measured repeatedly
☐	☒	The statistical test(s) used AND whether they are one- or two-sided<br>*Only common tests should be described solely by name; describe more complex techniques in the Methods section.*
☐	☒	A description of all covariates tested
☐	☒	A description of any assumptions or corrections, such as tests of normality and adjustment for multiple comparisons
☐	☒	A full description of the statistical parameters including central tendency (e.g. means) or other basic estimates (e.g. regression coefficient) AND variation (e.g. standard deviation) or associated estimates of uncertainty (e.g. confidence intervals)
☐	☒	For null hypothesis testing, the test statistic (e.g. *F*, *t*, *r*) with confidence intervals, effect sizes, degrees of freedom and *P* value noted<br>*Give P values as exact values whenever suitable.*
☒	☐	For Bayesian analysis, information on the choice of priors and Markov chain Monte Carlo settings
☒	☐	For hierarchical and complex designs, identification of the appropriate level for tests and full reporting of outcomes
☐	☒	Estimates of effect sizes (e.g. Cohen's *d*, Pearson's *r*), indicating how they were calculated

Our web collection on statistics for biologists contains articles on many of the points above.

Software and code

Policy information about availability of computer code

Data collection

Actimetry was measured with VersaMax Legacy Open Field activity box (Omnitech Electronics, In.). Behavioral tests were recorded with Basler Ace acA1300-60gm camera and either manually or semi-automatically annotated the following software: Ethovision XT 16 analysis software, ANY-MAZE v6.0.

Immunohistochemistry was assessed with Leica TCS SP5 (setting 1: 1024x1024, 40x oil immersion objective, step-size of 2.01, zoom 2.4; setting 2: 512x512, 63x oil immersion objective, step-size of 1.76, zoom 2.46).

Magnetic resonance imaging was performed using a 9.4 T horizontal bore magnet (Bruker BioSpin) with 12 cm wide actively shielded gra-dient coils (440 mT/m). Radiofrequency transmission was achieved with a birdcage volume resonator and signal was detected using a two-element arrayed surface coil (RAPID Biomedi-cal) position over the brain of the animal. MRI sequences: the protocol of the study consisted of covering the whole brain with T2-weighted image (T2-wi) in axial and coronal orientations. Also, T1-weighted images (T2-wi) were acquired in order to evaluate any possible artifacts in the images. A Rapid Acquisition with Refocused Echoes (RARE) sequence with the following parameters was used to obtain a T2-weighted anatomical image: 23 axial orientation slices, echo time (TE) = 11 ms, repetition time (TR) = 2.5 s, rare factor (RF) = 8, slice thickness 0.5 mm, no slice separation, field of view (FOV) = 20 x 20 mm2, matrix size 256 x 256 (isotropic in plane resolution of 0.078 mm/pixel). The RARE-T2 in coronal orientation has the same parameters with 16 slices. Images were processed using the software FIJI: ImageJ (v1.50i).

SWATH proteomics peptide identification was performed with the ProteinPilot software (v5.0.1, Sciex) in a joint search across the 10 runs. For the Paragon method, the following parameters were used: trypsin enzyme, iodoacetamide as alkylating agent, and the mouse UniProt proteome as reference (17/12/2021) with Sciex's contaminant database. Proteins were selected under an FDR significance level of 0.01. Finally, the data were preprocessed using PeakView (v2.2, Sciex) with the SWATH 2.0 microapp, generating total area-normalized

measurements for each protein.

(see Methods for extended details)

Data analysis	Visualization of results was carried out using IGV (v2.9.4), deepTools (v3.5.0) and the R packages ggplot2 (v3.3.5), ComplexHeatmap (v2.8.0), EnrichedHeatmap (v1.22.0), circlize (v0.4.10), GViz (v1.30.3), ComplexUpset (v1.3.3), eulerr (v6.1.0) and ggalluvial (v0.12.3).<br><br>Behavioral and immunohistochemical data were analyzed with the R packages: rstatix (v0.7.2), car (v3.1.2), rcompanion (v2.4.30).<br><br>RNA-seq data were processed with: fastp (v0.20.1). Transcript-level quantification was obtained using Salmon (v1.5.0). Further processing and analyses were performed within R, using: tximport (v1.14.2), biomaRt (v2.42.0), edgeR (v3.28.1), DESeq2 (v1.26.0), IsoformSwitchAnalyzeR (v1.8.0), MuSiC (v0.2.0), scRNAseq (v2.0.2).<br><br>EM-seq data were processed with: BCREval (v0), Trim Galore! (v0.6.4), Bismark (v0.22.2). Further processing and analyses were performed with metilene (v0.2.8) and using the R software.<br><br>ATAC-seq and ChIP-seq data were processed with: fastp (v0.20.1), Bowtie 2 (v2.4.2), Picard (v2.23.9), SAMtools (v1.7), BEDTools (v2.29.2), deepTools (v3.5.0), featureCounts (v2.0.1) and epic2 (v0.0.48). Further processing and analyses were performed within R, using: GreyListChIP (v1.18.0), Rsubread (v2.0.1), DESeq2 (v1.26.0). Chromatin state analyses were performed using ChromHMM (v1.23).<br><br>SWATH proteomics data were preprocessed with ProteinPilot software (v5.0.1, Sciex) and PeakView (v2.2, Sciex). Further processing and analyses were performed within R, using: biomaRt (v2.42.0), limma (v3.42.2).<br><br>Single cell Multiome data were processed with the Chromium Multiome Cell Ranger ARC pipeline (v2.0.1, 10X Genomics). Further processing and analyses were performed within R, using: Seurat (v4.3.0), SingleCellExperiment (v1.20.0), Signac (v1.8.0), scds (v1.14.0), scDblFinder (v1.12.0), Augur (v1.0.3).<br><br>Other bioinformatic analyses were performed using: LISA (lisa2 v0), msigdbr (v7.4.1), goseq (v1.38.0), GenomicRanges (v1.39.3), ChIPseeker (v1.22.1), regioneR (v1.18.1), LOLA (v1.16.0).<br><br>(see Methods for extended details)<br><br>All code underlying this study is publicly available at Zenodo: https://zenodo.org/doi/10.5281/zenodo.8372431.

For manuscripts utilizing custom algorithms or software that are central to the research but not yet described in published literature, software must be made available to editors and reviewers. We strongly encourage code deposition in a community repository (e.g. GitHub). See the Nature Portfolio guidelines for submitting code & software for further information.

Data

Policy information about availability of data

All manuscripts must include a data availability statement. This statement should provide the following information, where applicable:

- Accession codes, unique identifiers, or web links for publicly available datasets
- A description of any restrictions on data availability
- For clinical datasets or third party data, please ensure that the statement adheres to our policy

The data underpinning this article are available in the article and in its Supplementary Material. Additionally, the raw sequencing data have been deposited in the European Nucleotide Archive (ENA) under the following accession numbers: PRJEB58981 [https://www.ebi.ac.uk/ena/browser/view/PRJEB58981] (RNA-seq), PRJEB59326 [https://www.ebi.ac.uk/ena/browser/view/PRJEB59326] (EM-seq), PRJEB59328 [https://www.ebi.ac.uk/ena/browser/view/PRJEB59328] (ATAC-seq), PRJEB59330 [https://www.ebi.ac.uk/ena/browser/view/PRJEB59330] (ChIP-seq) and PRJEB59404 [https://www.ebi.ac.uk/ena/browser/view/PRJEB59404] (single cell RNA-seq and ATAC-seq). The raw proteomics data have been deposited in the Proteomics Identification Database (PRIDE) under the accession number PXD045567 [https://www.ebi.ac.uk/pride/archive/projects/PXD045567]. Finally, preprocessed and extended data sets, including chromatin state annotations and preprocessed single cell data (Seurat objects), are available in a Zenodo repository at https://zenodo.org/doi/10.5281/zenodo.8372431.

All code underlying this study is publicly available at Zenodo: https://zenodo.org/doi/10.5281/zenodo.8372431.

Research involving human participants, their data, or biological material

Policy information about studies with human participants or human data. See also policy information about sex, gender (identity/presentation), and sexual orientation and race, ethnicity and racism.

Reporting on sex and gender	No human subjects were used in this study.
Reporting on race, ethnicity, or other socially relevant groupings	No human subjects were used in this study.
Population characteristics	n/a
Recruitment	n/a

Ethics oversight	n/a

Note that full information on the approval of the study protocol must also be provided in the manuscript.

Field-specific reporting

Please select the one below that is the best fit for your research. If you are not sure, read the appropriate sections before making your selection.

☒ Life sciences ☐ Behavioural & social sciences ☐ Ecological, evolutionary & environmental sciences

For a reference copy of the document with all sections, see nature.com/documents/nr-reporting-summary-flat.pdf

Life sciences study design

All studies must disclose on these points even when the disclosure is negative.

Sample size	No statistical methods were used to pre-determine sample sizes. These were chosen to be similar to or larger than previous literature in the field.
Data exclusions	Data exclusion procedures were implemented to analyze the behavioral data due to the variability intrinsic to these types of tests. Outliers defined as Q3 +- 1.5xIQR were flagged and kept and extreme outliers defined as Q3 + 3xIQR were removed from the tests
Replication	Different numbers and types of replicates were used across the different experiments. For behavioral and immunohistochemical data, between 3 and 6 animals were used per group, and independent validation experiments were carried out in a different laboratory for EPM and NOL.<br><br>For sequencing experiments, RNA-seq used 3 biological replicates per group, and an independent RNA-seq experiment of similar conditions was carried out in an independent laboratory. For ChIP-seq and ATAC-seq, 2 biological replicates were used per group. For EM-seq, 1 biological replicate was used per group (pool of 3 different animals). For SWATH, 3 biological replicates were used per group, with each biological replicate each having 3 technical-replicate runs. For Single Cell Multiome, 1 biological replicate was used per group (pool of 3 different animals).<br><br>All attempts to repeat the experiments were successful. One replicate of the ChIP-seq immunoprecipitations failed after QC (H3K27ac for the young enriched group), so pseudotechnical replicates were used for that particular condition.
Randomization	Animals from each age group were randomly assigned to control or enriched groups.
Blinding	The investigators were not blinded during data collection of analyses. Blinding was not possible because the experimental design caused noticeable phenotypic differences among groups (e.g. old vs young).

Reporting for specific materials, systems and methods

We require information from authors about some types of materials, experimental systems and methods used in many studies. Here, indicate whether each material, system or method listed is relevant to your study. If you are not sure if a list item applies to your research, read the appropriate section before selecting a response.

Materials & experimental systems

n/a	Involved in the study	
☐	☒	Antibodies
☒	☐	Eukaryotic cell lines
☒	☐	Palaeontology and archaeology
☐	☒	Animals and other organisms
☒	☐	Clinical data
☒	☐	Dual use research of concern
☒	☐	Plants

Methods

n/a	Involved in the study	
☐	☒	ChIP-seq
☒	☐	Flow cytometry
☐	☒	MRI-based neuroimaging

Antibodies

Antibodies used	Primary antibodies used for immunohistochemistry were:<br><br>DCX, goat anti-doublecortin, 1:500, Santa Cruz #sc-8066;<br><br>CLR, rabbit anti-calretinin, 1:3000, Swant #7697;<br><br>SOX2, goat anti-sex determining region Y-box 2, 1:200, R&D Systems #AF2018;

GFAP, Rabbit anti-glial fibrillary acidic protein, 1:2000, Abcam #ab7260;

pH3, Rabbit anti-phospho-histone H3, 1:500, Millipore #06-570;

Secondary antibodies used for immunohistochemistry were:

Donkey anti-goat alexa fluor 594 #A-11058 Invitrogen;

Donkey anti-rabbit alexa fluor 594 #A-21207 Invitrogen;

Donkey anti-rabbit alexa fluor 488 #A-21206 Invitrogen;

Donkey anti-rat alexa fluor 594 #A-21209 Invitrogen;

For ChIP-seq, the following antibodies were used:

H3K4me3 (Diagenode, #C15410003, Lot. A1051D);

H3K4me1 (Diagenode, #C15410194, Lot. A1862D);

H3K27ac (Diagenode, #C15410196, Lot. A1723-0041D);

H3K9me3 (Diagenode, #C15410193, Lot. A0219P);

H3K27me3 (Diagenode, #C15410195, Lot. A0821D);

H3K36me3 (Diagenode, #C15410192, Lot. A1845P);

Validation

Primary antibodies used for immunohistochemistry were:

DCX, goat anti-doublecortin, 1:500, Santa Cruz #sc-8066; validated for IHC and used in at least 225 publications as stated on the product page (https://www.scbt.com/p/doublecortin-antibody-c-18?productCanUrl=doublecortin-antibody-c-18&_requestid=676799).

CLR, rabbit anti-calretinin, 1:3000, Swant #7697; vallidated for IHC and used in various publications as described in the product pages (https://www.swant.com/?p=products&c=1.2; https://www.labome.com/summary/SWant.html).

SOX2, goat anti-sex determining region Y-box 2, 1:200, R&D Systems #AF2018; validated for IHC and used in at least 196 publications as stated on the product page (https://www.rndsystems.com/products/human-mouse-rat-sox2-antibody_af2018).

GFAP, Rabbit anti-glial fibrillary acidic protein, 1:2000, Abcam #ab7260; validated for IHC and used in at least 1012 publications as stated on the product page (https://www.abcam.com/products/primary-antibodies/gfap-antibody-ab7260.html)

pH3, Rabbit anti-phospho-histone H3, 1:500, Millipore #06-570; validated for IHC and used in multiple publications as stated on the product page (https://www.merckmillipore.com/ES/es/product/Anti-phospho-Histone-H3-Ser10-Antibody-Mitosis-Marker,MM_NF-06-570)

Secondary antibodies used for immunohistochemistry were:

Donkey anti-goat alexa fluor 594 #A-11058 Invitrogen; validated for IHC and used in at least 1035 publications as stated on the product page (https://www.thermofisher.com/antibody/product/Donkey-anti-Goat-IgG-H-L-Cross-Adsorbed-Secondary-Antibody-Polyclonal/A-11058)

Donkey anti-rabbit alexa fluor 594 #A-21207 Invitrogen; validated for IHC and used in at least 2276 publications as stated on the product page (https://www.thermofisher.com/antibody/product/Donkey-anti-Rabbit-IgG-H-L-Highly-Cross-Adsorbed-Secondary-Antibody-Polyclonal/A-21207)

Donkey anti-rabbit alexa fluor 488 #A-21206 Invitrogen; validated for IHC and used in at least 6121 publications as stated on the product page (https://www.thermofisher.com/antibody/product/Donkey-anti-Rabbit-IgG-H-L-Highly-Cross-Adsorbed-Secondary-Antibody-Polyclonal/A-21206)

Donkey anti-rat alexa fluor 594 #A-21209 Invitrogen; validated for IHC and used in at least 568 publications as stated on the product page (https://www.thermofisher.com/antibody/product/Donkey-anti-Rat-IgG-H-L-Highly-Cross-Adsorbed-Secondary-Antibody-Polyclonal/A-21209)

For ChIP-seq, the following antibodies were used:

H3K4me3 (Diagenode, #C15410003, Lot. A1051D); validated for ChIP-seq and used in multiple publications as stated on the product page (https://www.diagenode.com/en/p/h3k4me3-polyclonal-antibody-premium-50-ug-50-ul)

H3K4me1 (Diagenode, #C15410194, Lot. A1862D); validated for ChIP-seq and used in multiple publications as stated on the product page (https://www.diagenode.com/en/p/h3k4me1-polyclonal-antibody-premium-50-mg#)

H3K27ac (Diagenode, #C15410196, Lot. A1723-0041D); validated for ChIP-seq and used in multiple publications as stated on the product page (https://www.diagenode.com/en/p/h3k27ac-polyclonal-antibody-premium-50-mg-18-ml)

H3K9me3 (Diagenode, #C15410193, Lot. A0219P); validated for ChIP-seq and used in multiple publications as stated on the product page (https://www.diagenode.com/en/p/h3k9me3-polyclonal-antibody-premium-50-mg)

H3K27me3 (Diagenode, #C15410195, Lot. A0821D); validated for ChIP-seq and used in multiple publications as stated on the product page (https://www.diagenode.com/en/p/h3k27me3-polyclonal-antibody-premium-50-mg-27-ml)

H3K36me3 (Diagenode, #C15410192, Lot. A1845P); validated for ChIP-seq and used in multiple publications as stated on the product page (https://www.diagenode.com/en/p/h3k36me3-polyclonal-antibody-premium-50-mg)

Animals and other research organisms

Policy information about studies involving animals; ARRIVE guidelines recommended for reporting animal research, and Sex and Gender in Research

Laboratory animals	Male C57BL/6JRj mice (Mus musculus) of 9 weeks and 17 months old were acquired from Janvier Labs.
Wild animals	The study did not involve wild animals.
Reporting on sex	All animals used were male. Male mice were used because of their longer lifespan which better suited the aging model.
Field-collected samples	The study did not involve animals collected from the field.
Ethics oversight	Mice were housed under standard laboratory conditions including ad libitum access to food and water, light/dark cycles (12h/12h) and stable temperature (20-22 ºC), in accordance with the European Union Directive 2010/63/EU.<br><br>All experiments were performed following the European Community Guidelines (Directive 2010/05/2016) and the Spanish Guidelines (Real Decreto 53/2013) for animal research, and were approved by the ethical committees of the Cajal Institute (Committee of Ethics and Animal Experimentation), the Spanish Research Council (Subcommittee of Ethics), the Animal Protection Area of the Ministry of Environment of the Community of Madrid (code: PROEX 222/16) and the Research Ethics Committee of the University of Oviedo (Subcommittee on animals and genetically modified organisms, code: PROAE 08/2021).

Note that full information on the approval of the study protocol must also be provided in the manuscript.

Plants

Seed stocks	*Report on the source of all seed stocks or other plant material used. If applicable, state the seed stock centre and catalogue number. If plant specimens were collected from the field, describe the collection location, date and sampling procedures.*
Novel plant genotypes	*Describe the methods by which all novel plant genotypes were produced. This includes those generated by transgenic approaches, gene editing, chemical/radiation-based mutagenesis and hybridization. For transgenic lines, describe the transformation method, the number of independent lines analyzed and the generation upon which experiments were performed. For gene-edited lines, describe the editor used, the endogenous sequence targeted for editing, the targeting guide RNA sequence (if applicable) and how the editor was applied.*
Authentication	*Describe any authentication procedures for each seed stock used or novel genotype generated. Describe any experiments used to assess the effect of a mutation and, where applicable, how potential secondary effects (e.g. second site T-DNA insertions, mosiacism, off-target gene editing) were examined.*

ChIP-seq

Data deposition

☒ Confirm that both raw and final processed data have been deposited in a public database such as GEO.

☐ Confirm that you have deposited or provided access to graph files (e.g. BED files) for the called peaks.

Data access links<br>*May remain private before publication.*	ENA primary accession PRJEB59330 ; ENA secondary accession ERP144377
Files in database submission	*Provide a list of all files available in the database submission.*
Genome browser session<br>(e.g. UCSC)	*Provide a link to an anonymized genome browser session for "Initial submission" and "Revised version" documents only, to enable peer review. Write "no longer applicable" for "Final submission" documents.*

Methodology

Replicates	2 biological replicates per condition. If the enrichment efficiencies were subsequently found to be disparate between the samples, biological replicates were replaced with pseudotechnical replicates.
Sequencing depth	All experiments were paired end with the following library sizes:

Sequencing depth

JC_R1_Input	49204929
JC_R2_Input	47144522
JC_R1_H3K9me3	70814810
JC_R2_H3K9me3	58683700
JC_R1_H3K4me3	35315818
JC_R2_H3K4me3	37879891
JC_R1_H3K4me1	41997334
JC_R2_H3K4me1	31046560
JC_R1_H3K36me3	56920253
JC_R2_H3K36me3	52803510
JC_R1_H3K27me3	56411164
JC_R2_H3K27me3	59884175
JC_R1_H3K27ac	19093090
JC_R2_H3K27ac	15697001
JE_R1_Input	49631685
JE_R2_Input	51446288
JE_R1_H3K9me3	46456894
JE_R2_H3K9me3	50045076
JE_R1_H3K4me3	43295613
JE_R2_H3K4me3	35641895
JE_R1_H3K4me1	36927533
JE_R2_H3K4me1	32954168
JE_R1_H3K36me3	43739450
JE_R2_H3K36me3	55011394
JE_R1_H3K27me3	49000690
JE_R2_H3K27me3	45171234
JE_R1_H3K27ac	18210597
JE_R2_H3K27ac	18209573
VC_R1_Input	65503297
VC_R2_Input	48848762
VC_R1_H3K9me3	52704317
VC_R2_H3K9me3	57248668
VC_R1_H3K4me3	35772249
VC_R2_H3K4me3	39436874
VC_R1_H3K4me1	32203495
VC_R2_H3K4me1	37826273
VC_R1_H3K36me3	58760716
VC_R2_H3K36me3	57020910
VC_R1_H3K27me3	61100732
VC_R2_H3K27me3	44979772
VC_R1_H3K27ac	14851867
VC_R2_H3K27ac	23487010
VE_R1_Input	45626026
VE_R2_Input	63387307
VE_R1_H3K9me3	53572427
VE_R2_H3K9me3	46040024
VE_R1_H3K4me3	37539675
VE_R2_H3K4me3	38914956
VE_R1_H3K4me1	39178457
VE_R2_H3K4me1	34816641
VE_R1_H3K36me3	54079004
VE_R2_H3K36me3	59862074
VE_R1_H3K27me3	53014925
VE_R2_H3K27me3	47285560
VE_R1_H3K27ac	16803749
VE_R2_H3K27ac	21925438

Note that one replicate of the ChIP-seq immunoprecipitations failed after QC (H3K27ac for the young enriched group), so pseudotechnical replicates were used for that particular condition.

Antibodies

For ChIP-seq, the following antibodies were used:

H3K4me3 (Diagenode, #C15410003, Lot. A1051D);

H3K4me1 (Diagenode, #C15410194, Lot. A1862D);

H3K27ac (Diagenode, #C15410196, Lot. A1723-0041D);

H3K9me3 (Diagenode, #C15410193, Lot. A0219P);

H3K27me3 (Diagenode, #C15410195, Lot. A0821D);

H3K36me3 (Diagenode, #C15410192, Lot. A1845P);

Peak calling parameters

Regions enriched in histone modifications (peaks) were called for each biological replicate using the epic2 reimplementation

Peak calling parameters	(v0.0.48) of SICER and optimized parameters for each histone mark:<br><br>--bin-size 100 --gaps-allowed 2 for sharp marks (H3K4me3);<br>--bin-size 200 --gaps-allowed 3 for narrow marks (H3K4me1, H3K27ac);<br>--bin-size 500 --gaps-allowed 4 for broad marks (H3K36me3, H3K27me3, H3K9me3);<br><br>All peaks were called with --false-discovery-rate-cutoff 0.05. The peaks of biological replicates were combined by taking the union of overlapping peaks.
Data quality	All reported peaks are above FDR 5% and retained if overlapping between the 2 replicates for each sample.
Software	FASTQ files were preprocessed and quality controlled with fastp (v0.20.1) using the following options: -l 20 -p --adapter_fasta. In brief, Illumina TruSeq adapter sequences were trimmed using a custom FASTA file and reads containing > 5 N nucleotides, reads containing > 40% proportion of < 15 Phred nucleotides and reads of < 20 bp in length were filtered out.<br>Next, reads were aligned to the indexed mm10 genome via Bowtie 2 (v2.4.2) with the options -X 2000 --very-sensitive-local.<br>Alignments were deduplicated using Picard (v2.23.9) and filtered using SAMtools (v1.7) to retain only properly paired reads with MAPQ >10 mapping to autosomal and sex chromosomes. Subsequently, ENCODE blacklisted regions (mm10,<br>v2) were filtered out from the alignments using BEDTools (v2.29.2). In addition, the Input control samples were used to define experiment-specific greylists with the R/Bioconductor packages GreyListChIP (v1.18.0) and BSgenome.Mmusculus.UCSC.mm10 (v1.4.0) by detecting regions of high and probably spurious signal in the Input samples. These regions were also removed from the alignments using BEDTools (v2.29.2). bigWig visualization files were generated at different resolutions with deepTools bamCoverage (v3.5.0) using the RPGC normalization.

Magnetic resonance imaging

Experimental design

Design type	Brain segmentation. Volume quantification
Design specifications	1 MRI study / animal
Behavioral performance measures	The following brain volumes regions were quantified: i) total brain, ii) cerebellum, iii) corpus callosum, iv) cerebral cortex: frontal lobe, v) cerebral cortex: parieto-temporal lobe, vi) hippocampal formation, vii) CA2 of hippocampus, viii) pre - para subiculum, and, ix) striatum.

Acquisition

Imaging type(s)	Structural
Field strength	9.4 T
Sequence & imaging parameters	The protocol of the study consisted of covering the whole brain with T2-weighted image (T2-wi) in axial and coronal orientations. Also, a T1-weighted image (T2-wi) were acquired in order to evaluate some possible artefacts in the images. A Rapid Acquisition with Refocused Echoes (RARE) sequence with the following parameters was used to obtain a T2-weighted anatomical image: 23 axial orientation slices, echo time (TE)= 11 ms, repetition time (TR)= 2.5 s, rare factor (RF)=8, slice thickness 0.5 mm, no slice separation, field of view (FOV)= 20 x 20 mm2, matrix size 256 x 256 (isotropic in plane resolution of 0.078 mm/pixel). The RARE-T2 in coronal orientation has the same parameters with 16 slices.
Area of acquisition	Whole Brain

Diffusion MRI ☐ Used ☒ Not used

Preprocessing

Preprocessing software	Images were processed, using the software FIJI: ImageJ (1.50i, Rasband W, NIH) ,and brain atlas:<br>Mouse Hippocampal atlas by Badhwar, Lerch, Hamel, Sled 2013 (https://scalablebrainatlas.incf.org/mouse).
Normalization	-
Normalization template	-
Noise and artifact removal	-
Volume censoring	-

Statistical modeling & inference

Model type and settings	Wilcoxon Rank SUm
Effect(s) tested	Volme differences

Specify type of analysis: ☐ Whole brain ☒ ROI-based ☐ Both

Anatomical location(s): i) total brain, ii) cerebellum, iii) corpus callosum, iv) cerebral cortex: frontal lobe, v) cerebral cortex: parieto-temporal lobe, vi) hippocampal formation, vii) CA2 of hippocampus, viii) pre - para subiculum, and, ix) striatum.

Statistic type for inference (See Eklund et al. 2016): -

Correction: -

Models & analysis

n/a	Involved in the study
☐	☐ Functional and/or effective connectivity
☐	☐ Graph analysis
☐	☐ Multivariate modeling or predictive analysis

Functional and/or effective connectivity: *Report the measures of dependence used and the model details (e.g. Pearson correlation, partial correlation, mutual information).*

Graph analysis: *Report the dependent variable and connectivity measure, specifying weighted graph or binarized graph, subject- or group-level, and the global and/or node summaries used (e.g. clustering coefficient, efficiency, etc.).*

Multivariate modeling and predictive analysis: *Specify independent variables, features extraction and dimension reduction, model, training and evaluation metrics.*
